# Supplementary material for: Neuro-cognitive specificities in prosocial disobedience: A comparative fMRI study of civilian and military populations
Source: PLoS One. 2025 Jul 22;20(7):e0328407. doi: 10.1371/journal.pone.0328407 (PMC12282893; doi:10.1371/journal.pone.0328407)
Supplement: S1 File — These analyses were conducted to investigate which individual characteristics, assessed by questionnaires, could best explain prosocial disobedience. (DOCX) [file pone.0328407.s001.docx]

**S1 File. Methodology and results of the linear regression analyses.**

These analyses were conducted to investigate which individual characteristics, assessed by questionnaires, could best explain prosocial disobedience.

Military participants completed the following questionnaires, at home, before the MRI session:

- the Aggression-Submission-Conventionalism (**ASC**) scale (Dunwoody & Funke, 2016)o investigate the relationship with authority, referring to the idea that some people need very little situational pressure to submit to an authority figure and to accept illegitimate orders to hurt others, while other individuals need significantly more.
- the Attitude for Money (**AfM**) scale (Yamauchi & Templer, 1982) to investigate if the impact of money reward obtained after each shock could be different according to the standard money attitude of each participant.
- the Moral Foundation (**MF**) questionnaire (Graham et al., 2011) to investigate the difference in sensitivity to distinct competing issues of morality.
- the Resistance to Peer Influence (**RPI**) scale (Steinberg & Monahan, 2007) to investigate how much people think another person can influence them.
- the Short Dark Triad (**SD3**) scale (Jones & Paulhus, 2014) to investigate three socially aversive traits: Machiavellianism, narcissism, and psychopathy.
- Interpersonal Reactivity Index (**IRI**) (Davis, 1980), to investigate subjective thoughts and feelings of empathy.

Immediately after the MRI session ended, military participants completed an additional questionnaire:

- the Social Identification with the Experimenter (**SocId**) questionnaire (Steffens et al., 2014) to investigate whether agents who identified most closely with the experimenter, were also more prone to follow the orders during the experiment.

Linear regression analyses were conducted as an exploratory analysis between the %Pro_disob and the questionnaires’ scores (ASC, AfM, MF, RPI, SD3, IRI, SocId). All sub-scores of each questionnaire were integrated in the analysis. We conducted both forward and backward stepwise regressions to select the best model according to the AIC, for each Agency and Empathy runs.

For the Agency run, the best model included nine factors (AIC=322.93): Submission of ASC (coef=6.00), Conventionalism of ASC (coef=-2.59), Harm of AfM (coef=1.75), Distrust of MF (coef=1.81), Anxiety of MF (coef=-1.74), RPI score (coef=16.38), Machiavellianism of SD3 (coef=1.83), Personal distress of IRI (coef=1.2) and Personal bound of SocId (coef=-1.6). The linear regression analysis on this model revealed a significant effect of all the factors (all t’s>|2.3|, all p’s<0.03), except the Conventionalism of ASC and the Personal distress of IRI (t’s<|1.76|, p’s>0.08). Two of these significant factors had a negative relationship with the %Pro_disob: Anxiety of MF and Personal bound of SocId.

For the Empathy run, the best model included eleven factors (AIC=303.79): Submission of ASC (coef=5.66), Conventionalism of ASC (coef=-4.61), Aggression of ASC (coef=-4.22), Purity of AfM (coef=1.63), Distrust of MF (coef=1.35), Anxiety of MF (coef=-1.41), RPI score (coef=15.18), Machiavellianism of SD3 (coef=1.3), Psychopathy of SD3 (coef=-1.39), Fantasy of IRI (coef=1.26) and Personal Identification of SocId (coef=-1.2). The linear regression analysis on this model revealed a significant (or marginal) effect of all factors (all t’s>|2|, all p’s<0.052), except the Psychopathy of SD3 (t=-1.74, p=0.09). Four of these significant factors had a negative relationship with the %Pro_disob: Conventionalism of ASC, Aggression of ASC, Anxiety of MF, Personal Identification of SocId.
