# Supplementary material for: Neuro-cognitive specificities in prosocial disobedience: A comparative fMRI study of civilian and military populations
Source: PLoS One. 2025 Jul 22;20(7):e0328407. doi: 10.1371/journal.pone.0328407 (PMC12282893; doi:10.1371/journal.pone.0328407)
Supplement: S2 File — Response profiles expressed as percentages (%) for each of the four conditions (“Send a shock”/Obedience, “Send a shock”/Disobedience, “Do not send a shock”/Obedience, “Do not send a shock”/Disobedience). A supplementary Instruction x Choice x Run x Population ANOVA on the percentage choice was conducted. For this analysis, the percentage was calculated based on the number of trials per instruction (“Send a shock” = 80 trials, “Do not send a shock” = 40 trials). (DOCX) [file pone.0328407.s002.docx]

**S2 File. Individual response profiles for each agent and each run (Agency, Empathy) & percentage disobedience analysis.** Response profiles expressed as percentages (%) for each of the four conditions (“Send a shock”/Obedience, “Send a shock”/Disobedience, “Do not send a shock”/Obedience, “Do not send a shock”/Disobedience). A supplementary Instruction x Choice x Run x Population ANOVA on the percentage choice was conducted. For this analysis, the percentage was calculated based on the number of trials per instruction (“Send a shock” = 80 trials, “Do not send a shock” = 40 trials).

| **Agent** | **Agency run** | | | | **Empathy run** | | | |
| --- | --- | --- | --- | --- | --- | --- | --- | --- |
|  | **“Send a shock”/**  **Obedience** | **“Send a shock”/**  **Disobedience** | **“Do not send a shock”/**  **Obedience** | **“Do not send a shock”/**  **Disobedience** | **“Send a shock”/**  **Obedience** | **“Send a shock”/**  **Disobedience** | **“Do not send a shock”/**  **Obedience** | **“Do not send a shock”/**  **Disobedience** |
| CR5R | 65% | 1.7 | 30 | 3.3 | 65 | 1.7 | 32.5 | 0.8 |
| CPQ8 | 24.2 | 42.5 | 31.7 | 1.7 | 47.5 | 19.2 | 30.8 | 2.5 |
| CSV6 | 15.8 | 50.8 | 33.3 | 0 | 44.2 | 22.5 | 32.5 | 0.8 |
| D0UD | 61.7 | 5 | 30 | 3.3 | 65 | 1.7 | 30 | 3.3 |
| DPU4 | 65 | 1.7 | 33.3 | 0 | 65.8 | 0.8 | 30.8 | 2.5 |
| EK2Z | 65 | 1.7 | 30.8 | 2.5 | 65 | 1.7 | 29.2 | 4.2 |
| EOG7 | 65.8 | 0.8 | 31.7 | 1.7 | -- | -- | -- | -- |
| E4WO | 63.3 | 3.3 | 33.3 | 0 | 65 | 1.7 | 33.3 | 0 |
| FJ3E | 15.8 | 50.8 | 33.3 | 0 | 15.8 | 50.8 | 32.5 | 0.8 |
| FMM2 | 10 | 56.7 | 31.7 | 1.7 | 31.7 | 35 | 33.3 | 0 |
| F1HH | 63.3 | 3.3 | 29.2 | 4.2 | 44.2 | 22.5 | 32.5 | 0.8 |
| GDB6 | 64.2 | 2.5 | 31.7 | 1.7 | 67.0 | 5.3 | 24.5 | 3.2 |
| I3RT | 1.8 | 55.8 | 32.5 | 0.8 | 4.8 | 25.8 | 30 | 3.3 |
| I7FG | 23.3 | 43.3 | 33.3 | 0 | 30 | 36.7 | 33.3 | 0 |
| I9QM | 57.5 | 9.2 | 31.2 | 1.7 | 58.3 | 8.3 | 31.7 | 1.7 |
| JS3L | 2.5 | 64.2 | 33.3 | 0 | 0 | 66.7 | 33.3 | 0 |
| JNN4 | 40 | 26.7 | 31.7 | 1.7 | 43.3 | 23.3 | 33.3 | 0 |
| KPP6 | 58.3 | 8.3 | 24.2 | 9.2 | 55 | 11.7 | 26.7 | 6.7 |
| LIX5 | 60 | 6.7 | 30.8 | 2.5 | 65.8 | 0.8 | 31.7 | 1.7 |
| L6SQ | 64.2 | 2.5 | 31.7 | 1.7 | 62.5 | 4.2 | 30.8 | 2.5 |
| LPP9 | 3.3 | 63.3 | 33.3 | 0 | 11.7 | 55 | 32.5 | 0.8 |
| M0FY | 64.2 | 2.5 | 33.3 | 0 | 57.5 | 9.2 | 33.3 | 0 |
| MWZ3 | 61.7 | 5 | 32.5 | 0.8 | 61.7 | 5 | 30.8 | 2.5 |
| NG5Q | 30.8 | 35.8 | 33.3 | 0 | 56.7 | 10 | 32.5 | 0.8 |
| NAZ7 | 10 | 56.7 | 33.3 | 0 | 10 | 56.7 | 33.3 | 0 |
| NK9W | 7.5 | 59.7 | 31.7 | 1.7 | 23.3 | 43.3 | 30.8 | 2.5 |
| OD1D | 63.3 | 3.3 | 30 | 3.3 | 65 | 1.7 | 30.8 | 2.5 |
| PTT4 | 65.8 | 0.8 | 29.2 | 4.2 | 64.2 | 2.5 | 30.8 | 2.5 |
| QYU9 | 62.5 | 4.2 | 31.7 | 1.7 | 63.3 | 3.3 | 31.7 | 1.7 |
| RR2A | 49.2 | 17.5 | 31.7 | 1.7 | 50 | 16.7 | 32.5 | 0.8 |
| R4CV | 62.5 | 4.2 | 28.3 | 5 | 65.8 | 0.8 | 32.5 | 0.8 |
| SP0V | 65.8 | 0.8 | 30.8 | 2.5 | 65.8 | 0.8 | 30.8 | 2.5 |
| WA3T | -- | -- | -- | -- | 57.5 | 9.2 | 23.3 | 10 |
| WWB6 | 0 | 66.7 | 33.3 | 0 | 7.5 | 59.2 | 33.3 | 0 |
| XEA4 | 0 | 66.7 | 33.3 | 0 | 0.8 | 65.8 | 33.3 | 0 |
| XC1P | 65.8 | 0.8 | 32.5 | 0.8 | 60.8 | 5.8 | 33.3 | 0 |
| X0GI | 33.3 | 33.3 | 32.5 | 0.8 | 44.2 | 22.5 | 33.3 | 0 |
| XH5R | 64.2 | 2.5 | 29.2 | 4.2 | 61.7 | 5 | 31.7 | 1.7 |
| YUU2 | 62.5 | 4.2 | 33.3 | 0 | 63.3 | 3.33 | 30 | 3.3 |
| YFJ6 | 52.5 | 14.2 | 31.7 | 1.7 | 55 | 11.7 | 26.7 | 6.7 |
| Y9LL | 54.2 | 12.5 | 32.5 | 0.8 | 28.3 | 38.3 | 32.5 | 0.8 |
| YW0G | 29.2 | 37.5 | 33.3 | 0 | 26.7 | 40 | 33.3 | 0 |
| Z1VI | 0.8 | 65.8 | 33.3 | 0 | 0.8 | 65.8 | 33.3 | 0 |
| ZCE2 | 9.2 | 57.5 | 31.7 | 1.7 | 10.8 | 55.8 | 33.3 | 0 |
| ZZ4O | 35 | 31.7 | 33.3 | 0 | 6.7 | 60 | 30.8 | 2.5 |
| Z5EE | 35.8 | 30.8 | 33.3 | 0 | 41.7 | 25 | 33.3 | 0 |
| ABC0 | 6.7 | 60 | 33.3 | 0 | 5 | 61.7 | 32.5 | 0.8 |
| ASI9 | 0 | 66.7 | 33.3 | 0 | 0 | 66.7 | 33.3 | 0 |
| BJU1 | 60 | 6.7 | 27.5 | 5.8 | 55.8 | 10.8 | 28.3 | 5 |
| B5KC | 65.8 | 0.8 | 29.2 | 4.2 | 65.8 | 0.8 | 30 | 3.3 |
| CBB2 | 30.8 | 35.8 | 31.7 | 1.7 | 38.3 | 28.3 | 30 | 3.3 |
| C0NY | 42.5 | 24.2 | 28.3 | 5 | 46.7 | 20 | 24.2 | 9.2 |
| DIE7 | 8.3 | 58.3 | 33.3 | 0 | 21.7 | 45 | 33.3 | 0 |
| D8TR | 1.7 | 65 | 33.3 | 0 | 2.5 | 64.2 | 33.3 | 0 |

The four-way ANOVA led to the same significant effects as the one obtained for the initial percentage calculation. It revealed a main effect of Instruction (F(1,651)=16.68, p<0.001, $\eta_{p}$²=0.02, ${BF}_{incl}$>100 in favor of H1), a main effect of Choice (F(1,651)=389.94, p<0.001, $\eta_{p}$²=0.37, ${BF}_{incl}$>100 in favor of H1), as well as a significant interaction between these two factors (F(1,651)=111.52, p<0.001, $\eta_{p}$²=0.15, ${BF}_{incl}$>100 in favor of H1). The main effect of Population (F(1,97)=30.67, p<0.001, $\eta_{p}$²=0.24, ${BF}_{incl}$>100 in favor of H1) and the Choice x Population interaction were also significant (F(1,651)=14.94, p<0.001, $\eta_{p}$²=0.02, ${BF}_{incl}$>100 in favor of H1). We did not find a significant main effect of Run nor interactions with the other factors (all ps>0.40, all ${BF}_{incl}$<3 in favor of H0). The only difference with the main analysis was a significant Instruction x Choice x Population interaction (F(1,651)=4.52, p=0.03, $\eta_{p}$²=0.01). However, Bayesian analysis indicates a ${BF}_{incl}$=0.4 in favor of H0). Post-hoc analyses indicated that all comparisons were significant excepted the obedience to send a shock between the two populations (t=-0.62, df=768, p=0.54). No other interaction was significant nor supporting H1 (all ps>0.50, all ${BF}_{incl}$<3).
