# Supplementary figures and images for: Neuro-cognitive specificities in prosocial disobedience: A comparative fMRI study of civilian and military populations
Source: PLoS One. 2025 Jul 22;20(7):e0328407. doi: 10.1371/journal.pone.0328407 (PMC12282893; doi:10.1371/journal.pone.0328407)

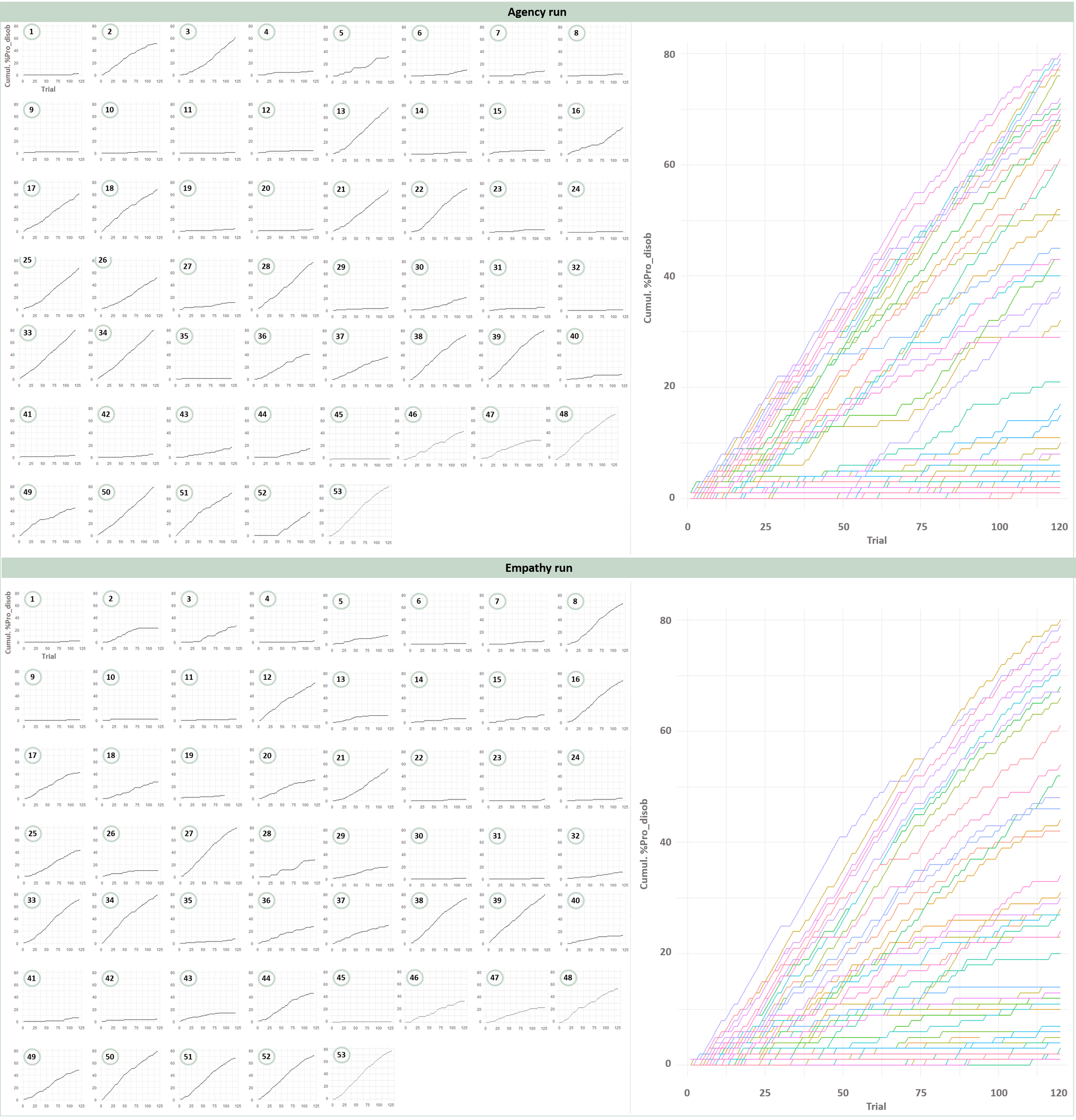

Supplement: S1 Fig — Individual plots (left side) and group plots (right side) representing the cumulative %Pro_disob (up to 80, as the instruction to send a shock was given 80 times) over the 120 trials for both Agency (top) and Empathy (bottom) runs. Participants were generally consistent across the two runs in their decision-making strategies. (TIF) [file pone.0328407.s003.tif]

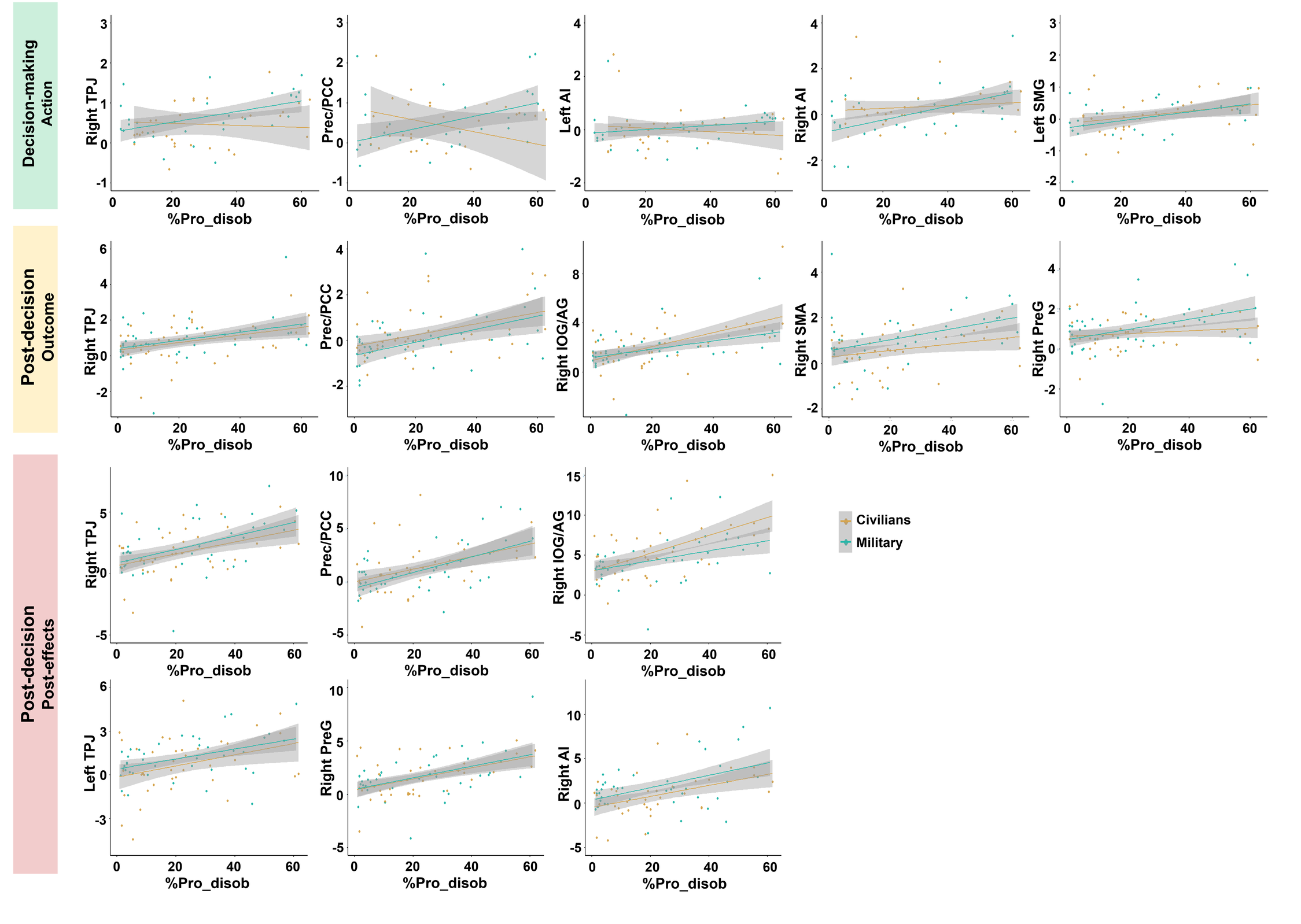

Supplement: S2 Fig — Segmented regression model analysis between the ROIs and %Pro_disob, taking the Population factor (Civilians, Military) into account. These plots concern the ROI’s activity ~ %Pro_disob x Population model. An effect of Population was only found for the decision-making phase, suggesting that the positive relationship between %Pro_disob and the right TPJ, Prec/PCC, and bilateral AI, was stronger in military participants than civilians. (TIF) [file pone.0328407.s004.tif]
