# Supplementary material for: Neuro-cognitive specificities in prosocial disobedience: A comparative fMRI study of civilian and military populations
Source: PLoS One. 2025 Jul 22;20(7):e0328407. doi: 10.1371/journal.pone.0328407 (PMC12282893; doi:10.1371/journal.pone.0328407)
Supplement: S3 File — A: Segmented regression model analysis between the subjective feelings (feeling of responsibility, feeling of guilt, feeling of sadness, perception of shock’s painfulness) and %Pro_disob, taking the Population factor (Civilians, Military) into account. Except for the feeling of responsibility, a Population effect was found for the majority of the investigated models suggesting that the positive relationship between %Pro_disob and the subjective feelings, was stronger in military participants than civilians. These plots concern the Subjective measure ~ %Pro_disob x Population model. B: Segmented regression model analysis between the disobedience criteria (morality, sensitivity, education) and %Pro_disob, taking the Population factor (Civilians, Military) into account. A Population effect was only found for sensitivity criterion suggesting that the positive relationship between %Pro_disob and the sensitivity criterion was stronger in military than civilians. Morality and education criteria appeared to be positively associated with %Pro_disob in a similar way between civilians and military participants. These plots concern the Subjective measure ~ %Pro_disob x Population model. (DOCX) [file pone.0328407.s005.docx]

**S3 File. Segmented regression model analyses with questionnaires.**

A: Segmented regression model analysis between the subjective feelings (feeling of responsibility, feeling of guilt, feeling of sadness, perception of shock’s painfulness) and %Pro_disob, taking the Population factor (Civilians, Military) into account. Except for the feeling of responsibility, a Population effect was found for the majority of the investigated models suggesting that the positive relationship between %Pro_disob and the subjective feelings, was stronger in military participants than civilians. These plots concern the Subjective measure ~ %Pro_disob x Population model.

B: Segmented regression model analysis between the disobedience criteria (morality, sensitivity, education) and %Pro_disob, taking the Population factor (Civilians, Military) into account. A Population effect was only found for sensitivity criterion suggesting that the positive relationship between %Pro_disob and the sensitivity criterion was stronger in military than civilians. Morality and education criteria appeared to be positively associated with %Pro_disob in a similar way between civilians and military participants. These plots concern the Subjective measure ~ %Pro_disob x Population model.

*
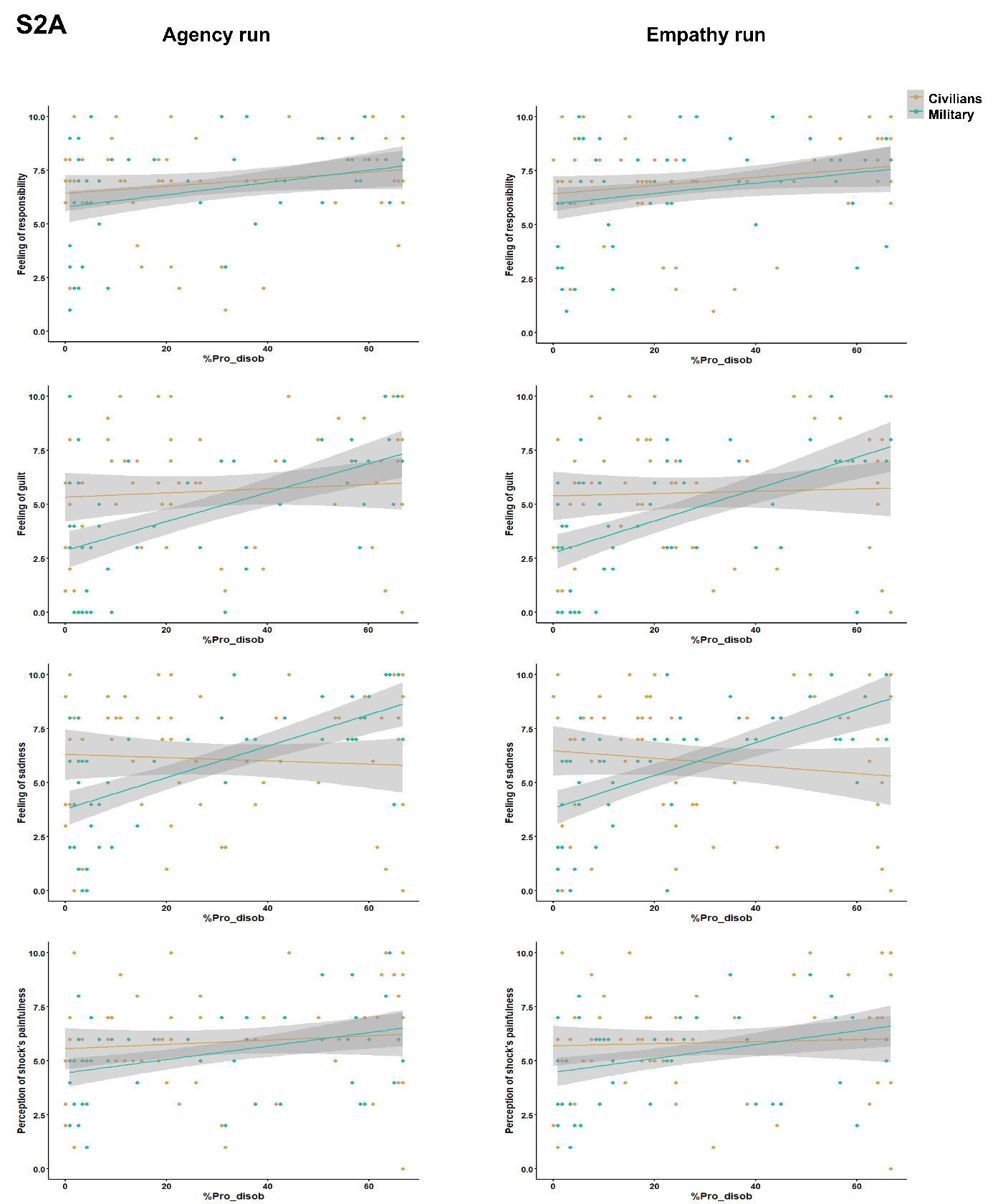

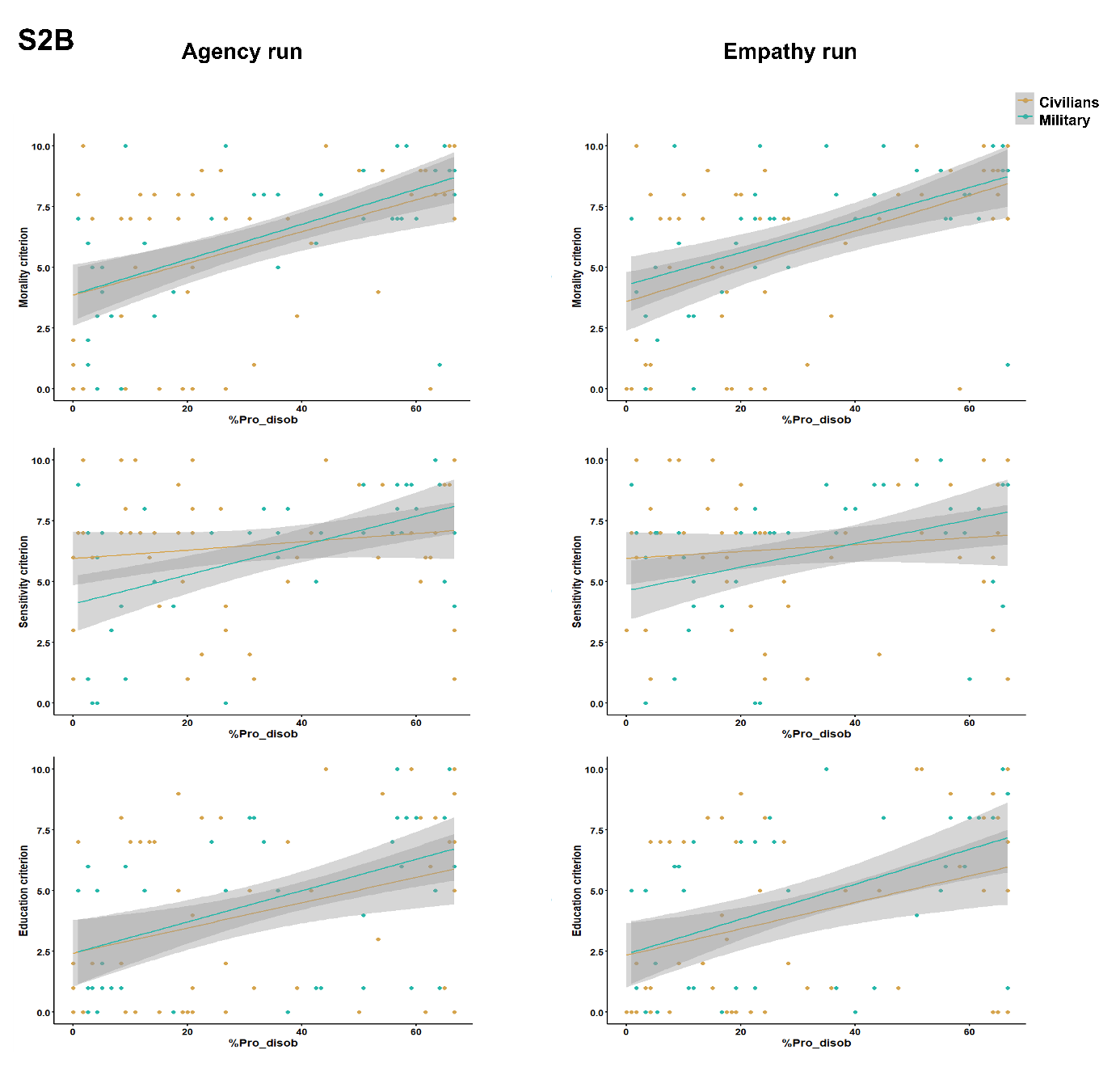
*
