# Supplementary material for: Neuro-cognitive specificities in prosocial disobedience: A comparative fMRI study of civilian and military populations
Source: PLoS One. 2025 Jul 22;20(7):e0328407. doi: 10.1371/journal.pone.0328407 (PMC12282893; doi:10.1371/journal.pone.0328407)

**S4 File. fMRI results at the whole-brain level for military participants.**

We used four contrasts of interest: 1) the [“Send a shock” > “Do not send a shock”] instruction contrast, 2) the [“Send a shock”/Disobedience > “Send a shock”/Obedience] decision type contrast, 3) the [“Send a shock”/Obedience > “Do not send a shock”/Obedience] obedience contrast and 4) the [“Send a shock”/Obedience + “Do not send a shock”/Disobedience] > [“Do not send a shock”/Obedience + “Send a shock”/Disobedience] outcome contrast. For the second contrast we also applied an exclusive mask using the significant clusters obtained from the [“Send a shock”/Obedience > “Do not send a shock”/Obedience] contrast. This mask filtered out all significant voxels identified in the [“Send a shock”/Obedience > “Do not send a shock”/Obedience] contrast, to retain only voxels representing brain activity associated with disobedience in the [“Send a shock”/Disobedience > “Send a shock”/Obedience] contrast. The significance threshold was set at p<0.05 (FWE corrected for multiple comparisons) at the cluster level, with an initial voxel-wise probability threshold of p<0.001 uncorrected; except for the analysis using the exclusive mask which was reported with a threshold of p<0.005 uncorrected.

*Pre-decision phase and auditory processing*

For this phase, we used the 3,000ms time window delay after the auditory instruction and before the keypress, during the Agency run. Using the [“Send a shock” > “Do not send a shock”] instruction contrast we found a significant activity in bilateral Inferior Occipital Gyrus extending to Angular Gyrus (IOG/AG), Temporo-Parietal Junction (TPJ), Inferior Temporal Gyrus (ITG) and right Supramarginal Gyrus (SMG) (Table S5A and Figure S5A). The reverse contrast revealed bilateral activation of the auditory areas (left: [-61 -13 6], Z = Inf., cluster size = 1542; right: [61 -9 3], Z = 7.50, cluster size = 1174), an effect likely driven by the fact that the “Do not send a shock” instruction was longer to process than “Send a shock” instruction (Figure S5A).

We used the [“Send a shock”/Disobedience > “Send a shock”/Obedience] contrast to investigate the changes between obedience and disobedience after an immoral order received. Significant clusters were only found for the reverse [“Send a shock”/Obedience > “Send a shock”/Disobedience] contrast, with a modulation of bilateral IOG/AG (Table S5B and Figure S5B). By applying the exclusive mask, we obtained three clusters: bilateral AG extending to the occipital gyrus and the Prec on the right hemisphere ([30 -74 36], Z = 3.57, cluster size = 417) and to the temporal gyrus on the left hemisphere ([-40 -61 9], Z = 3.69, cluster size = 284), and a cluster including the AI and the putamen ([23 12 -16], Z = 3.50, cluster size = 561).

Similar activity in IOG/AG but extending to the SMG toward the front and to the superior and middle occipital gyrus (SOG/MOG) toward the back, was found when comparing obedience to shock with obedience to no shock (Table S5C and Figure S5C). The reverse contrast showed the bilateral auditory areas (left: [-61 -16 9], Z = 7.24, cluster size = 1037; right: [61 -9 3], Z = 6.89, cluster size = 722), in line with the results explained above.

*Decision-making phase and action processing*

The decision-making phase was investigated during the Agency run with an epoch taken between the screen display of the Shock/No shock mapping, where participants could see which button was associated with which action, and the tone associated to the action’s outcome. Using the main [“Send a shock”/Obedience > “Send a shock”/Disobedience] contrast we found a significant activation of bilateral IOG/AG extending to the TPJ and SMG, as well as the left ITG extending to the Fusiform Gyrus and the left Lingual Gyrus (LingG) (Table S5B and Figure S5B). Even though larger, this cluster overlapped with the one found for the pre-decision phase. No significant cluster was found for the reverse contrasts. By applying the exclusive mask, we obtained two clusters: the left posterior part of the temporal gyrus extending toward SMG ([-49 -47 -22], Z = 4.09, cluster size = 255), and the right anterior part of the temporal gyrus ([27 0 -22], Z = 4.08, cluster size = 249).

For the [“Send a shock”/Obedience > “Do not send a shock”/Obedience] contrast similar clusters (IOG/AG, TPJ, SMG, temporal gyrus, LingG) were found but adding the vmPFC/ACC and the temporal pole, particularly involving the AI. No significant cluster emerged for the reverse contrast.

We also identified clusters of the [“Send a shock”/Obedience + “Do not send a shock”/Disobedience] > [“Do not send a shock”/Obedience + “Send a shock”/Disobedience] contrast to investigate brain differences when agents decided to send a shock to the victim compared to when they did not. We found higher activity in bilateral IOG/AG extending to TPJ, SMG and in the Temporal Gyrus, but also in the Precuneus extending to the Posterior Cingulate Cortex (Prec/PCC), and in the ventro-median Prefrontal Cortex (vMPFC) extending to Anterior Cingulate Cortex (ACC), as well as an orbital region (Table S5D and Figure S5D). No significant cluster was found for the reverse contrast.

*Post-decision phase and outcome processing*

We targeted the post-decision phase for outcome processing during the Empathy run with a 1,500ms time window corresponding to the display of the arrow pointing to the video of the victim’s hand receiving or not receiving the shock. We analyzed the [“Send a shock”/Obedience + “Do not send a shock”/Disobedience] > [“Do not send a shock”/Obedience + “Send a shock”/Disobedience] contrast to isolate the experience of witnessing the victim’s pain or not, and we obtained large clusters including bilateral IOG/AG, TPJ, STG, SMG and Superior Parietal Lobule (SPL), but also bilateral Precentral Gyrus (PreG), Precentral Sulcus (PreS) and Anterior Insula (AI) extending to the Inferior and Middle Frontal Gyrus (IFG/MFG), Supplementary Motor Area (SMA), and the Thalamus (Table S5D and Figure S5D). No significant cluster was found for the reverse contrast. The same network was obtained by comparing obedience to send a shock or no shock (Table S5C and Figure S5C). The reverse contrast revealed one significant cluster on the left AG ([-45 -76 45], Z = 4.36, cluster size = 177). Results obtained using the main [“Send a shock”/Obedience > “Send a shock”/Disobedience] contrast showed a similar network but could not be interpreted, as it involved different visible outcomes (i.e., a shock vs no shock) based on different decisions (i.e., obey/disobey) that could not be dissociated.

*Post-decision phase and post-effects*

In both Agency and Empathy runs, during the post-decision phase we also investigated post-effects using the time window between the tone or the arrow display and the scale display (i.e., the interval estimate scale or the pain rating scale). Seeing the victim receiving a shock compared to not receiving it activated the same regions as the ones observed and described above for the outcome processing: bilateral IOG/AG extending to TPJ, STG, SMG and SPL, bilateral PreG/PreS extending toward the anterior part with MFG, IFG, orbital region and AI. Medial regions were also found: SMA, dorso-median Prefrontal Cortex (dmPFC), Prec and Amydgala (Table S5D and Figure S5D). With the exception of the amygdala, the same clusters were found for [“Send a shock”/Obedience > “Do not send a shock”/Obedience] (Table S5C and Figure S5C). No significant cluster was found for the two reverse contrasts. Similar to the outcome processing, the network observed for the [“Send a shock”/Obedience > “Send a shock”/Disobedience] contrast could not be interpreted, but involved the same regions (IOG/AG, TPJ, SMG, left SPL and right IFG).

| **TABLE S5A** | | | | | |
| --- | --- | --- | --- | --- | --- |
| Anatomical location | MNI coordinates (in mm) | | | Z score | Cluster size  (number of voxels) |
| [“Send a shock” > “Do not send a shock”] | x | y | z |  |  |
| ***Pre-decision phase for auditory processing*** |  |  |  |  |  |
|  |  |  |  |  |  |
| Left Inferior Occipital Gyrus / Angular Gyrus / Temporo-Parietal Junction / Inferior Temporal Gyrus | -49 | -74 | 6 | 5.41 | 579 |
| Right Inferior Occipital Gyrus / Angular Gyrus / Temporo-Parietal Junction / Inferior Temporal Gyrus | 48 | -70 | 3 | 5.20 | 535 |
| Left Inferior Temporal Gyrus / Fusiform Gyrus | -47 | -38 | -16 | 4.67 | 147 |
| Right Supramarginal Gyrus | 57 | -29 | 33 | 3.91 | 93 |
|  |  |  |  |  |  |

| **TABLE S5B** | | | | | |
| --- | --- | --- | --- | --- | --- |
| Anatomical location | MNI coordinates (in mm) | | | Z score | Cluster size  (number of voxels) |
| “Send a shock”/Obedience > “Send a shock”/Disobedience | x | y | z |  |  |
| ***Pre-decision phase for auditory processing*** | | |  |  |  |
|  |  |  |  |  |  |
| Left Inferior Occipital Gyrus / Angular Gyrus | -47 | -67 | 6 | 4.78 | 578 |
| Right Inferior Occipital Gyrus / Angular Gyrus | 52 | -67 | 3 | 5.22 | 515 |
|  |  |  |  |  |  |
| ***Decision-making phase for action*** |  |  |  |  |  |
|  |  |  |  |  |  |
| Left Inferior Occipital Gyrus / Angular Gyrus / Temporo-Parietal Junction | -49 | -70 | 15 | 6.11 | 974 |
| Right Inferior Occipital Gyrus / Angular Gyrus / Temporo-Parietal Junction / Superior Temporal Gyrus / Supramarginal Gyrus | 48 | -65 | 9 | 5.73 | 1139 |
| Left Inferior Temporal Gyrus / Fusiform Gyrus | -45 | -45 | -22 | 5.19 | 149 |
| Left Lingual Gyrus | -7 | -79 | -4 | 4.09 | 105 |
|  |  |  |  |  |  |

| **TABLE S5C** | | | | | |
| --- | --- | --- | --- | --- | --- |
| Anatomical location | MNI coordinates (in mm) | | | Z score | Cluster size  (number of voxels) |
| “Send a shock”/Obedience > “Do not send a shock”/Obedience | x | y | z |  |  |
| ***Pre-decision phase for auditory processing*** | | |  |  |  |
|  |  |  |  |  |  |
| Left Inferior Occipital Gyrus / Angular Gyrus | -49 | -72 | 3 | 6.99 | 1155 |
| Right Inferior Occipital Gyrus / Angular Gyrus | 50 | -70 | 3 | 6.74 | 1175 |
| Left Superior and Middle Occipital Gyrus | -25 | -81 | 33 | 3.86 | 109 |
|  |  |  |  |  |  |
|  |  |  |  |  |  |
| ***Decision-making phase for action*** |  |  |  |  |  |
|  |  |  |  |  |  |
| Left Inferior Occipital Gyrus / Angular Gyrus / Temporo-Parietal Junction / Superior Temporal Gyrus | -52 | -72 | 9 | 7.39 | 4340 |
| Right Inferior and Middle Occipital Gyrus / Angular Gyrus / Temporo-Parietal Junction / Superior Temporal Gyrus / Supramarginal Gyrus / Precuneus / Posterior Cingulate Cortex / Lingual Gyrus | 50 | -70 | -4 | 7.40 | 3204 |
| Left Supramarginal Gyrus | -67 | -36 | 27 | 4.07 | 101 |
| Ventro-median Prefrontal Cortex / Anterior Cingulate Cortex | 0 | 59 | -10 | 4.82 | 243 |
| Right Temporal Pole / Anterior Insula | 43 | 18 | -19 | 4.44 | 117 |
|  |  |  |  |  |  |
|  |  |  |  |  |  |
| ***Post-decision phase for outcome*** |  |  |  |  |  |
|  |  |  |  |  |  |
| Left Inferior Occipital Gyrus / Angular Gyrus / Temporo-Parietal Junction / Superior Temporal Gyrus / Supramarginal Gyrus / Superior Parietal Lobule | -49 | -74 | 6 | 7.30 | 3287 |
| Right Inferior Occipital Gyrus / Angular Gyrus / Temporo-Parietal Junction / Superior Temporal Gyrus / Supramarginal Gyrus / Superior Parietal Lobule | 52 | -67 | -1 | 6.84 | 1735 |
| Left Precentral Gyrus / Precentral Sulcus / Inferior Frontal Gyrus / Anterior Insula / Orbital region | -34 | 27 | -1 | 6.08 | 587 |
| Left Precentral Gyrus / Middle Frontal Gyrus | -45 | -2 | 39 | 4.25 | 149 |
| Right Precentral Gyrus / Precentral Sulcus / Inferior Frontal Gyrus / Middle Frontal Gyrus / Anterior Insula / Orbital region | 50 | 23 | -7 | 6.95 | 2866 |
| Left Middle Frontal Gyrus | -29 | 48 | 30 | 4.07 | 162 |
| Supplementary Motor Area | 7 | 34 | 39 | 6.07 | 1352 |
|  |  |  |  |  |  |
|  |  |  |  |  |  |
| ***Post-decision phase for post-effects*** |  |  |  |  |  |
|  |  |  |  |  |  |
| Left Inferior Occipital Gyrus / Angular Gyrus / Temporo-Parietal Junction / Superior Temporal Gyrus / Supramarginal Gyrus / Superior Parietal Lobule | -52 | -72 | 6 | 7.33 | 3541 |
| Right Inferior Occipital Gyrus / Angular Gyrus / Temporo-Parietal Junction / Superior Temporal Gyrus / Supramarginal Gyrus / Superior Parietal Lobule | 52 | -67 | -1 | 7.34 | 3225 |
| Left Angular Gyrus / Supramarginal Gyrus / Superior Parietal Lobule | -34 | -52 | 48 | 4.12 | 153 |
| Left Precentral Gyrus / Precentral Sulcus / Inferior Frontal Gyrus / Anterior Insula / Orbital region | -36 | 27 | -1 | 5.46 | 861 |
| Right Precentral Gyrus / Precentral Sulcus / Inferior Frontal Gyrus / Anterior Insula / Orbital region / Supplementary Motor Area / dorso-median Prefrontal Cortex | 57 | 12 | 9 | 5.53 | 4739 |
| Left Middle Frontal Gyrus | -38 | 52 | 24 | 4.77 | 261 |
| Left Precuneus / Lingual Gyrus | -11 | -72 | 39 | 4.63 | 288 |
| Right Precuneus / Lingual Gyrus | 9 | -70 | 39 | 4.21 | 137 |
| Posterior Cingulate Cortex | 3 | -43 | 21 | 4.33 | 131 |

| **TABLE S5D** | | | | | |
| --- | --- | --- | --- | --- | --- |
| Anatomical location | MNI coordinates (in mm) | | | Z score | Cluster size  (number of voxels) |
| [“Send a shock”/Obedience + “Do not send a shock”/Disobedience] > [“Do not send a shock”/Obedience + “Send a shock”/Disobedience] | x | y | z |  |  |
| ***Decision-making phase for action*** |  |  |  |  |  |
|  |  |  |  |  |  |
| Left Inferior Occipital Gyrus / Angular Gyrus / Temporo-Parietal Junction / Supramarginal Gyrus / Precuneus / Posterior Cingulate Cortex / Temporal Gyrus | -52 | -72 | 9 | 7.46 | 4771 |
| Left Inferior Occipital Gyrus / Angular Gyrus / Temporo-Parietal Junction / Supramarginal Gyrus / Precuneus / Posterior Cingulate Cortex / Temporal Gyrus | 50 | -70 | -4 | 7.38 | 3056 |
| Anterior Cingulate Cortex / ventro-median Prefrontal Cortex | -2 | 43 | -7 | 4.69 | 284 |
| Orbital region | 36 | 32 | -19 | 4.50 | 106 |
|  |  |  |  |  |  |
|  |  |  |  |  |  |
| ***Post-decision phase for outcome*** |  |  |  |  |  |
|  |  |  |  |  |  |
| Left Inferior Occipital Gyrus / Angular Gyrus / Temporo-Parietal Junction / Superior Temporal Gyrus / Supramarginal Gyrus / Superior Parietal Lobule | -49 | -74 | 6 | 7.65 | 4460 |
| Right Inferior Occipital Gyrus / Angular Gyrus / Temporo-Parietal Junction / Superior Temporal Gyrus / Supramarginal Gyrus / Superior Parietal Lobule | 52 | -67 | -1 | 7.54 | 2969 |
| Left Precentral Gyrus / Precentral Sulcus / Inferior Frontal Gyrus / Anterior Insula / Orbital region | -34 | 27 | -1 | 6.33 | 1244 |
| Right Precentral Gyrus / Precentral Sulcus / Inferior Frontal Gyrus / Middle Frontal Gyrus / Anterior Insula / Orbital region | 52 | 21 | -7 | 6.79 | 3188 |
| Supplementary Motor Area | 5 | 34 | 39 | 5.82 | 1544 |
| Left Middle Frontal Gyrus | -29 | 43 | 24 | 4.75 | 263 |
| Thalamus | 5 | -2 | 6 | 4.34 | 222 |
|  |  |  |  |  |  |
|  |  |  |  |  |  |
| ***Post-decision phase for post-effects*** |  |  |  |  |  |
|  |  |  |  |  |  |
| Left Inferior Occipital Gyrus / Angular Gyrus / Temporo-Parietal Junction / Superior Temporal Gyrus / Supramarginal Gyrus / Superior Parietal Lobule | -52 | -72 | 6 | 7.24 | 4637 |
| Right Inferior Occipital Gyrus / Angular Gyrus / Temporo-Parietal Junction / Superior Temporal Gyrus / Supramarginal Gyrus / Superior Parietal Lobule | 52 | -67 | -1 | 7.33 | 3410 |
| Left Precentral Gyrus / Precentral Sulcus / Inferior Frontal Gyrus / Anterior Insula / Orbital region | -36 | 27 | -1 | 5.22 | 823 |
| Right Precentral Gyrus / Precentral Sulcus / Inferior Frontal Gyrus / Anterior Insula / Orbital region / Supplementary Motor Area / dorso-median Prefrontal Cortex | 54 | 36 | 6 | 5.46 | 4997 |
| Left Middle Frontal Gyrus | -38 | 52 | 24 | 4.79 | 321 |
| Left Precentral Gyrus | -49 | 0 | 42 | 4.34 | 123 |
| Right Precuneus | 9 | -70 | 39 | 4.22 | 124 |
| Left Amygdala | -27 | -2 | -19 | 4.41 | 160 |


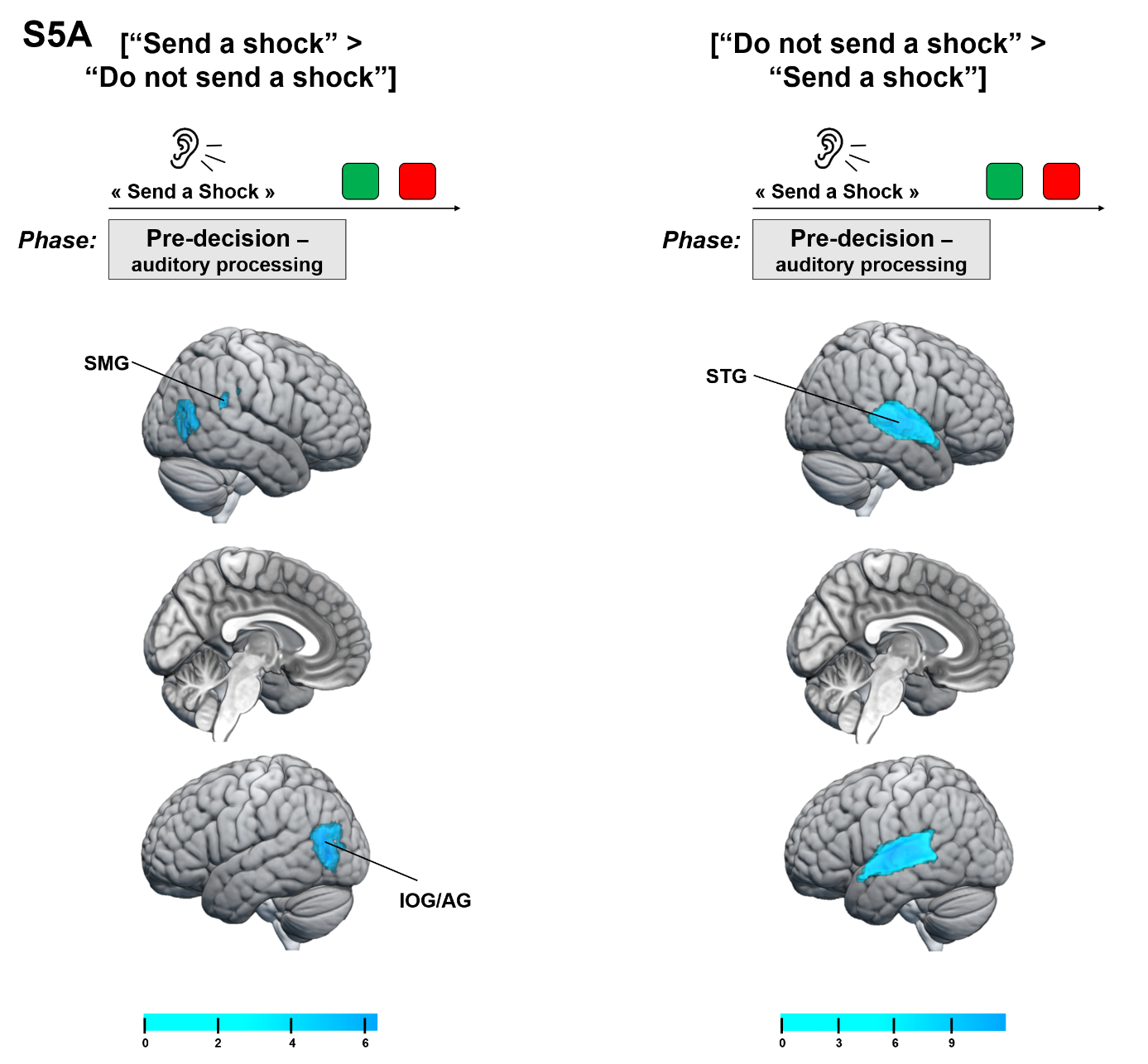


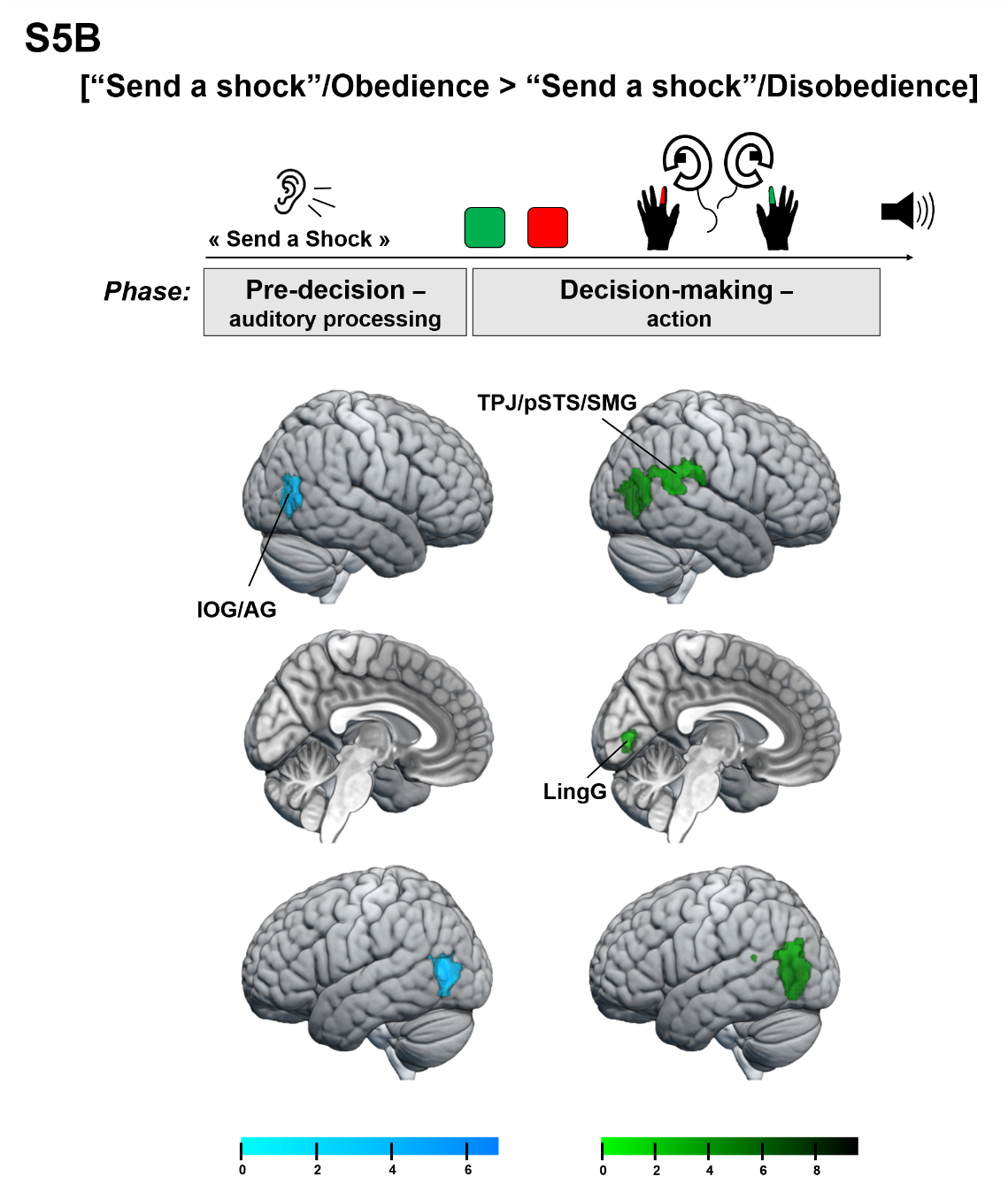


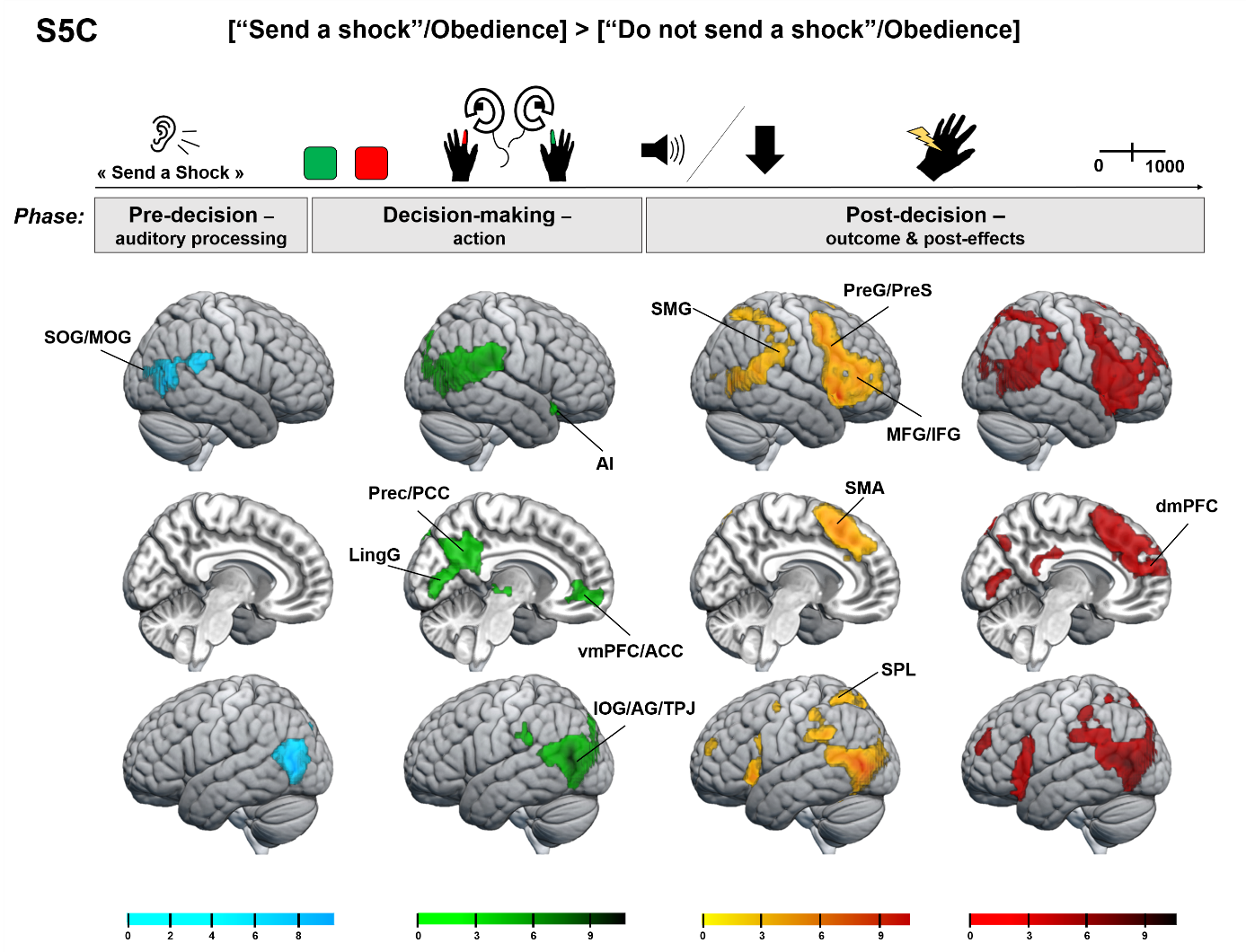


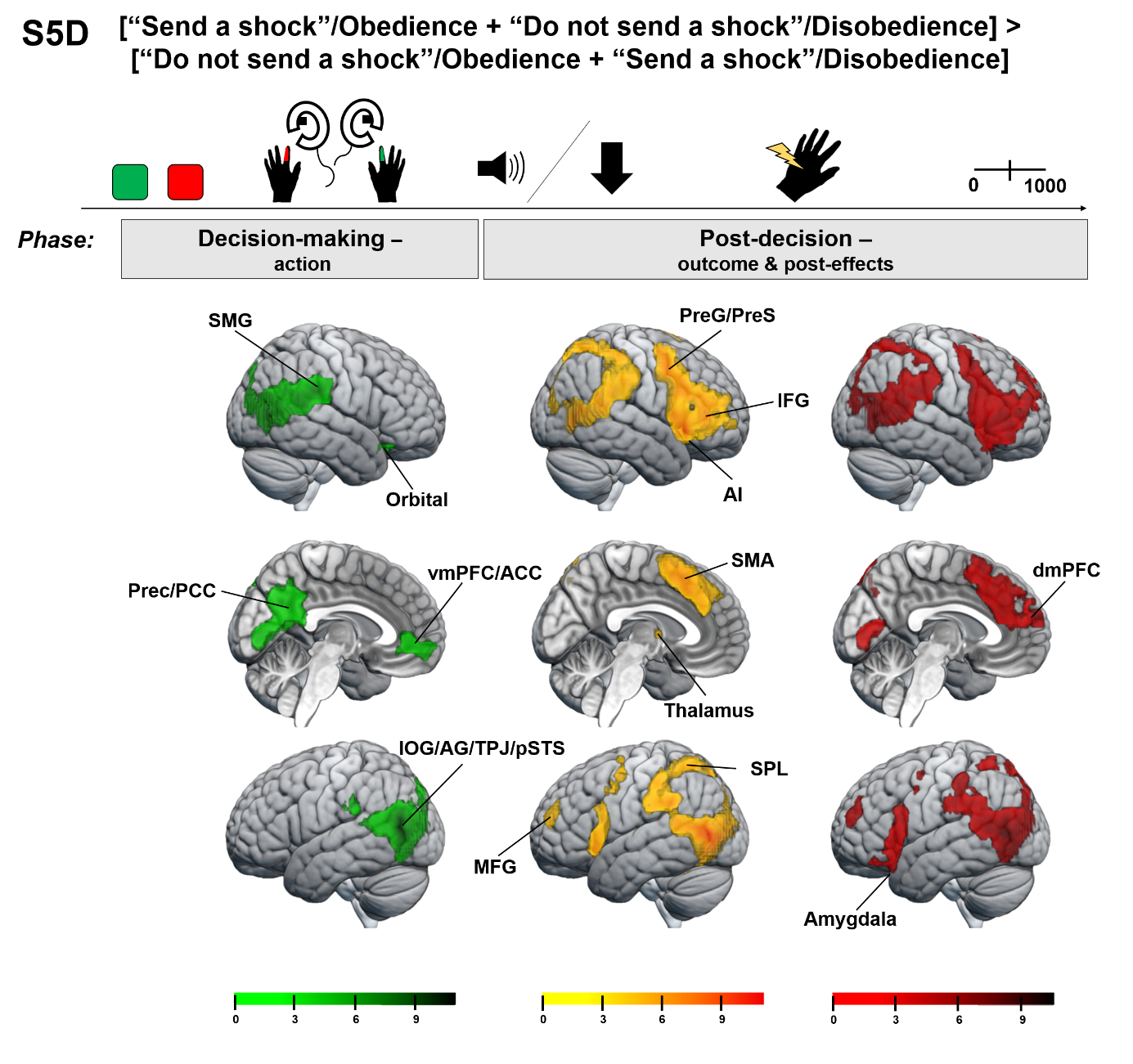

Supplement: S4 File — We used four contrasts of interest: 1) the [“Send a shock” > “Do not send a shock”] instruction contrast, 2) the [“Send a shock”/Disobedience > “Send a shock”/Obedience] decision type contrast, 3) the [“Send a shock”/Obedience > “Do not send a shock”/Obedience] obedience contrast and 4) the [“Send a shock”/Obedience + “Do not send a shock”/Disobedience]> [“Do not send a shock”/Obedience + “Send a shock”/Disobedience] outcome contrast. For the second contrast we also applied an exclusive mask using the significant clusters obtained from the [“Send a shock”/Obedience > “Do not send a shock”/Obedience] contrast. This mask filtered out all significant voxels identified in the [“Send a shock”/Obedience > “Do not send a shock”/Obedience] contrast, to retain only voxels representing brain activity associated with disobedience in the [“Send a shock”/Disobedience > “Send a shock”/Obedience] contrast. The significance threshold was set at p < 0.05 (FWE corrected for multiple comparisons) at the cluster level, with an initial voxel-wise probability threshold of p < 0.001 uncorrected; except for the analysis using the exclusive mask which was reported with a threshold of p < 0.005 uncorrected. (DOCX) [file pone.0328407.s006.docx]
