# Supplementary material for: Neuro-cognitive specificities in prosocial disobedience: A comparative fMRI study of civilian and military populations
Source: PLoS One. 2025 Jul 22;20(7):e0328407. doi: 10.1371/journal.pone.0328407 (PMC12282893; doi:10.1371/journal.pone.0328407)
Supplement: S5 File — Conjunction maps between military participants and civilians for the three other contrasts of interest: [“Send a shock”/Obedience > “Do not send a shock”/Obedience] (Fig A), [“Send a shock” > “Do not send a shock”] (Fig B) and [“Send a shock”/Obedience + “Do not send a shock”/Disobedience]> [“Do not send a shock”/Obedience + “Send a shock”/Disobedience] (Fig C). The first contrast focusing on obedience trials by contrasting the order received, was investigated for all time windows. The reverse contrast did not reveal any clusters. The contrast [“Send a shock” > “Do not send a shock”] and its reverse contrast, investigating the processing of the auditory instructions, were only applied during the pre-decision phase. Finally, the contrast comparing trials where the victim received pain or no pain, was studied between the decision-making and the post-decision periods. The reverse contrast did not reveal any clusters. (DOCX) [file pone.0328407.s007.docx]

**S5 File. Conjunction maps.**

Conjunction maps between military participants and civilians for the three other contrasts of interest: [“Send a shock”/Obedience > “Do not send a shock”/Obedience] (Fig A), [“Send a shock” > “Do not send a shock”] (Fig B) and [“Send a shock”/Obedience + “Do not send a shock”/Disobedience] > [“Do not send a shock”/Obedience + “Send a shock”/Disobedience] (Fig C). The first contrast focusing on obedience trials by contrasting the order received, was investigated for all time windows. The reverse contrast did not reveal any clusters. The contrast [“Send a shock” > “Do not send a shock”] and its reverse contrast, investigating the processing of the auditory instructions, were only applied during the pre-decision phase. Finally, the contrast comparing trials where the victim received pain or no pain, was studied between the decision-making and the post-decision periods. The reverse contrast did not reveal any clusters.

*
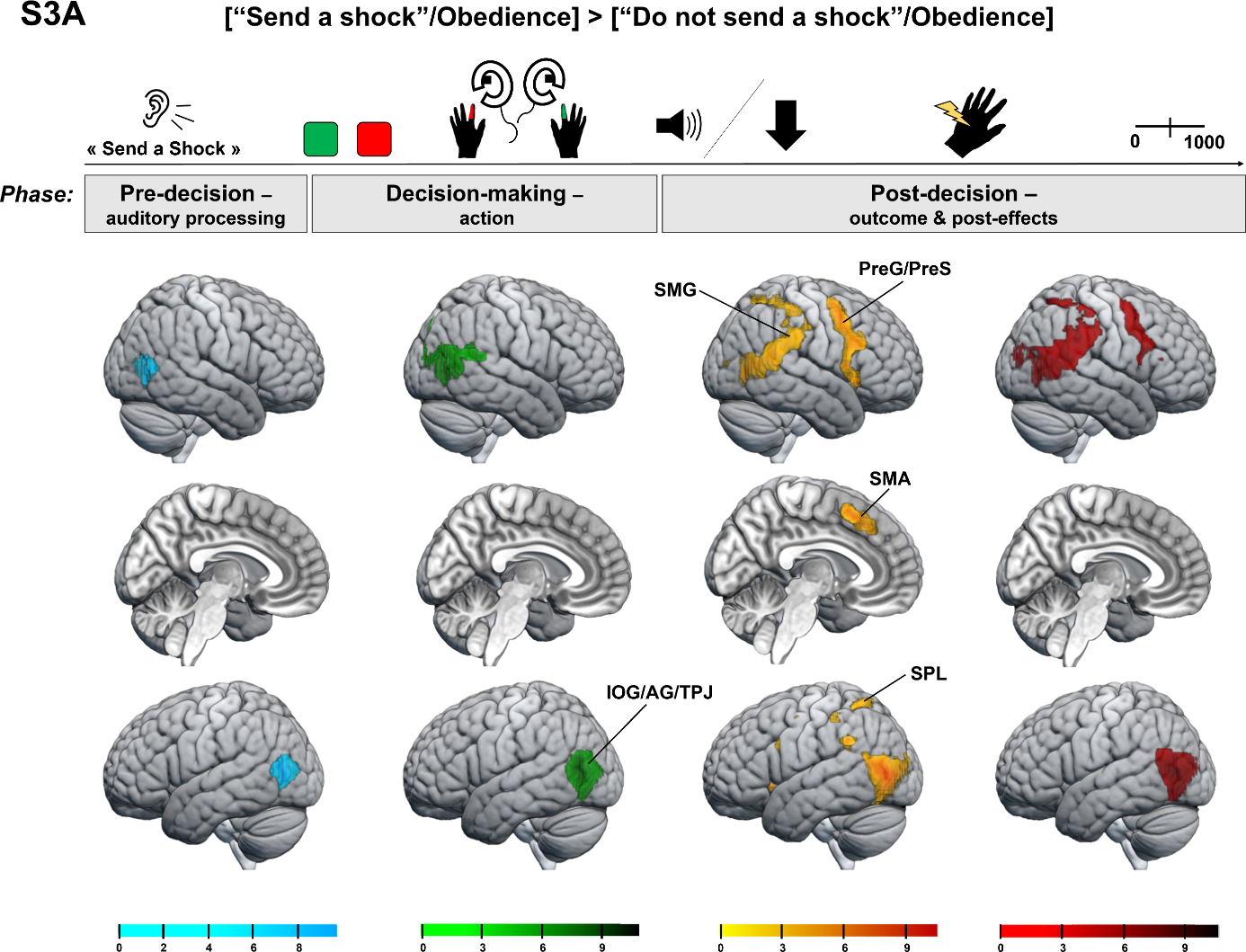

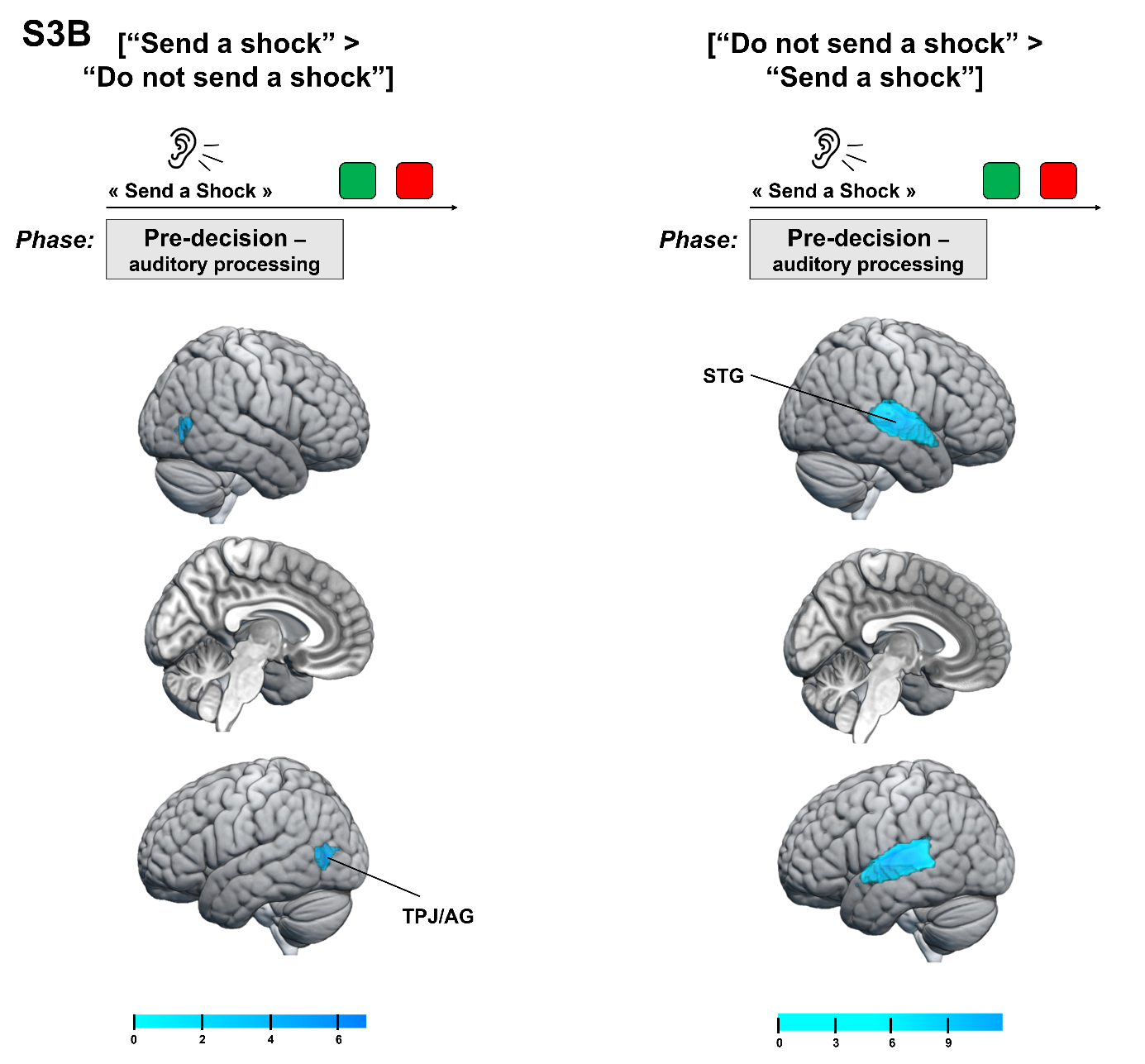

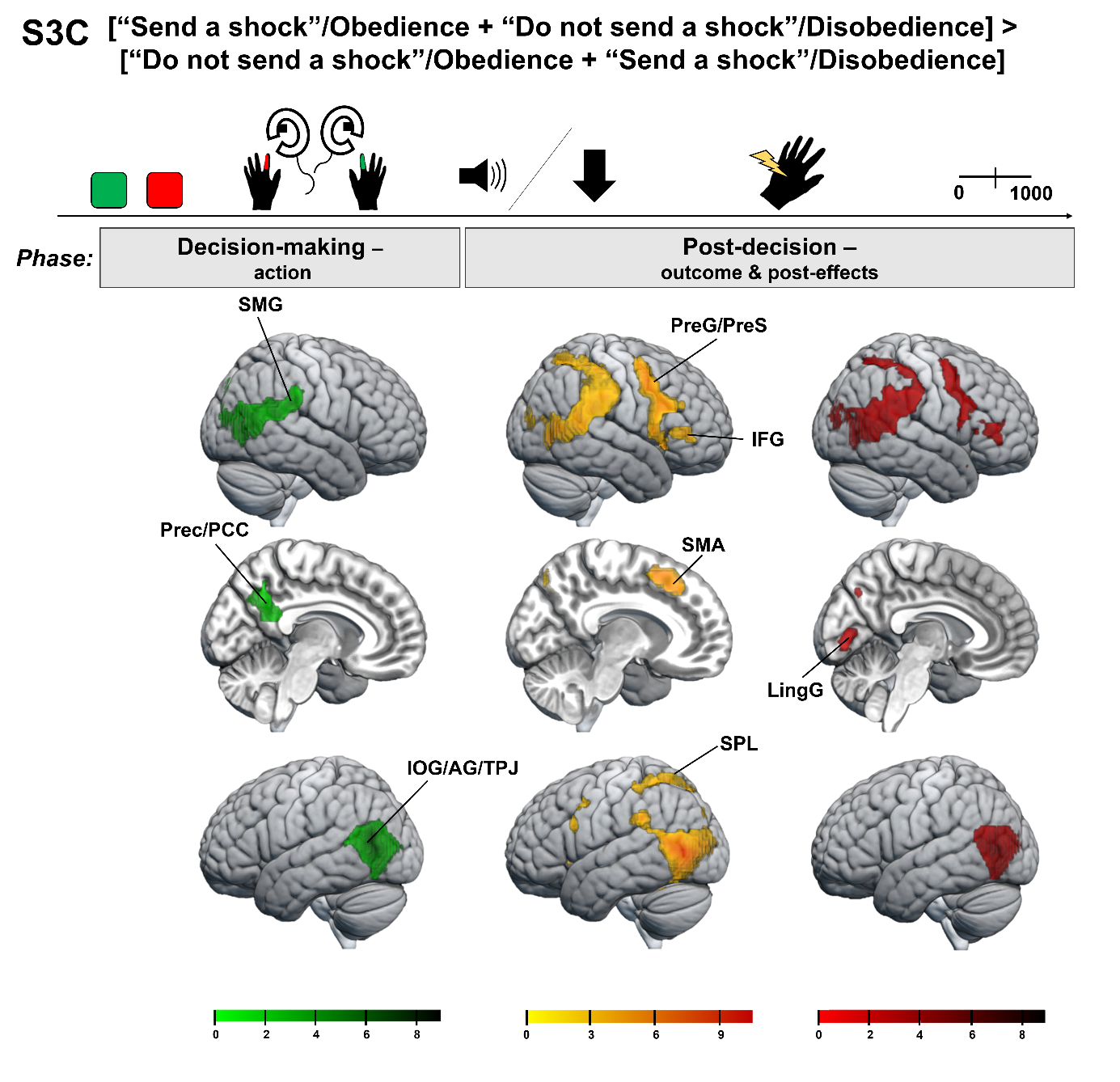
*
