# Supplementary material for: Neuro-cognitive specificities in prosocial disobedience: A comparative fMRI study of civilian and military populations
Source: PLoS One. 2025 Jul 22;20(7):e0328407. doi: 10.1371/journal.pone.0328407 (PMC12282893; doi:10.1371/journal.pone.0328407)
Supplement: S6 File — Segmented regression model analysis between the ROIs (i.e., bilateral AI, right TPJ and Prec/PCC) and disobedience criteria (i.e., shock quantity, money, entertainment, rejection of authority, experimental, fear of judgment, and country’s history criterions), taking the Population factor (Civilians, Military) into account. Both models are considered: ROI’s activity ~ criterion x Population (model 1) and criterion ~ ROI’s activity x Population (model 2). (DOCX) [file pone.0328407.s008.docx]

**S6 File. Segmented regression model analyses between disobedience’s criteria, significant ROIs, and population.**

Segmented regression model analysis between the ROIs (i.e., bilateral AI, right TPJ and Prec/PCC) and disobedience’s criteria (i.e., shock quantity, money, entertainment, rejection of authority, experimental, fear of judgment, and country’s history criteria), taking the Population factor (Civilians, Military) into account. Both models are considered: ROI’s activity ~ criterion x Population (model 1) and criterion ~ ROI’s activity x Population (model 2).

Shock quantity, fear of judgment, entertainment and money criteria did not show significant relationship with ROIs activities, nor interaction with Population (all p’s>0.05).

Country’s history criterion showed a stronger positive relationship with right TPJ and Prec/PCC activities in military officer cadets than civilians, particularly for model 1 (Model 1: p=0.01 for right TPJ and p=0.009 for Prec/PCC; Model 2: p=0.08 for right TPJ, p=0.06 for Prec/PCC).

Rejection of authority criterion showed a stronger positive relationship with left AI, right TPJ and Prec/PCC activities in military officer cadets than civilians (Model 1: p=0.01 for left AI, p=0.01 for right TPJ, p=0.005 for Prec/PCC; Model 2: p=0.02 for left AI, p=0.09 for right TPJ, p=0.04 for Prec/PCC).

Finally, for the experimental criterion only a significant main effect emerged showing that left AI activity negatively influenced this criterion (Model 1: p=0.04), with no interaction with the Population (all others p’s>0.08).
