# Supplementary material for: Neuro-cognitive specificities in prosocial disobedience: A comparative fMRI study of civilian and military populations
Source: PLoS One. 2025 Jul 22;20(7):e0328407. doi: 10.1371/journal.pone.0328407 (PMC12282893; doi:10.1371/journal.pone.0328407)

**S7 File. Segmented regression model analyses with no significant ROIs.**

Segmented regression model analysis between the ROIs and %Pro_disob, taking the Population factor (Civilians, Military) into account. Both models are considered: ROI’s activity ~ %Pro_disob x Population (model 1) and %Pro_disob ~ ROI’s activity x Population (model 2). Only ROIs that showed no significant correlation with %Pro_disob for the military participants are analyzed below (results on the remaining ROIs are described in the main manuscript).

Pre-decision phase (investigated using the [“Send a shock”/Obedience > “Send a shock”/Disobedience] contrast): A significant interaction Population x ROI’s activity on the %Pro_disob was found on the left AI (p=0.03) and dmPFC (p=0.049) with a tendency for the bilateral TPJ (p=0.08 for both sides). The Population x %Pro_disob interaction on ROI’s activity was only significant for the right TPJ (p=0.03) with a tendency for left TPJ (p=0.08) and dmPFC (p=0.08). These results indicated a negative relationship between the %Pro_disob and the activity in bilateral TPJ, that was stronger for civilians than for military participants. By contrast, the relationship between the %Pro_disob and the activity in dmPFC was positive, and stronger for civilians than military. A different pattern emerged for the left AI, with a positive relationship for both populations but at a distinct activity’s threshold (see figure A below).

Decision-making phase (investigated using the [“Send a shock”/Obedience > “Send a shock”/Disobedience] contrast): Only a Population effect on the two directions of the relationship was observed between the %Pro_disob and the left TPJ (model 1: p=0.050, model 2: p=0.02). It suggested a stronger positive relationship between %Pro_disob and left TPJ for military than civilians (figure B).

Post-decision phase targeting outcomes (investigated using the [“Send a shock”/Obedience > “Do not send a shock”/Obedience] contrast): No significant Population effect was found, whatever the ROIs and the used model.

Post-decision phase targeting post-effects (investigated using the [“Send a shock”/Obedience > “Do not send a shock”/Obedience] contrast): We found a significant Population x %Pro_disob on the activity of the left SMG (p=0.04) and dmPFC (p=0.005), remaining marginal for the left SPL (p=0.056). Model 2 showed only a significant relationship with the vmPFC/ACC (p=0.053). All results indicated a stronger positive relationship between %Pro_disob and left SMG, dmPFC, left SPL and vmPFC/ACC for civilians than military (figure C).

*Only significant results are showed on the figures.*


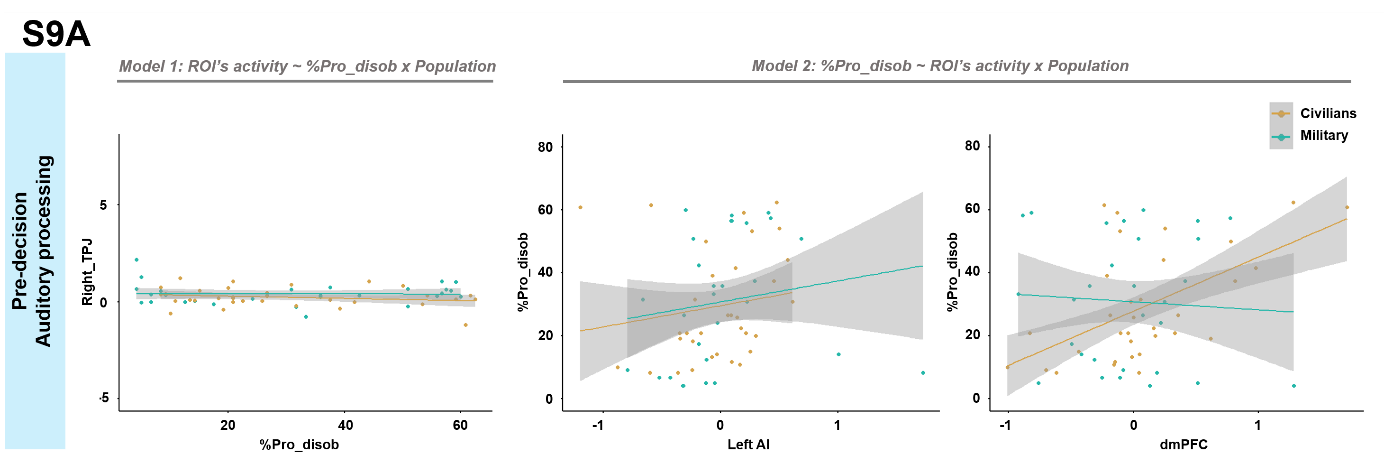


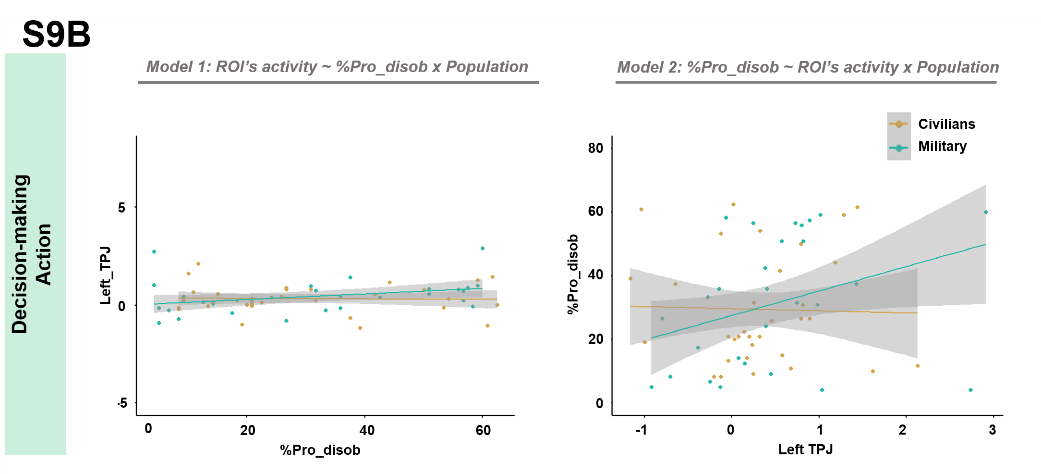


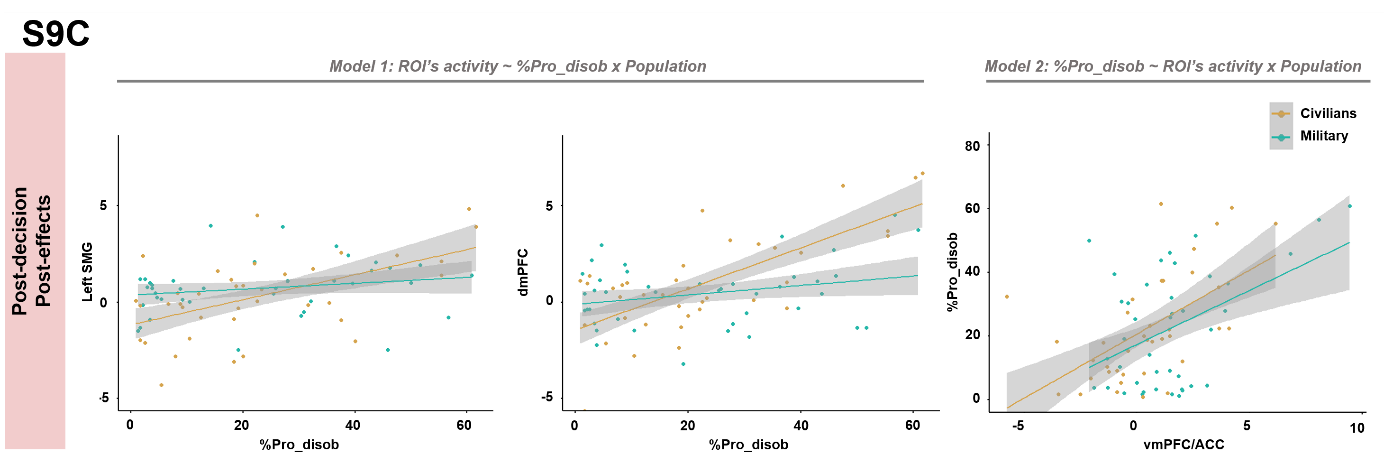

Supplement: S7 File — Segmented regression model analysis between the ROIs and %Pro_disob, taking the Population factor (Civilians, Military) into account. Both models are considered: ROI’s activity ~ %Pro_disob x Population (model 1) and %Pro_disob ~ ROI’s activity x Population (model 2). Only ROIs that showed no significant correlation with %Pro_disob for the military participants are analyzed below (results on the remaining ROIs are described in the main manuscript). (DOCX) [file pone.0328407.s009.docx]
