# Supplementary material for: Neuro-cognitive specificities in prosocial disobedience: A comparative fMRI study of civilian and military populations
Source: PLoS One. 2025 Jul 22;20(7):e0328407. doi: 10.1371/journal.pone.0328407 (PMC12282893; doi:10.1371/journal.pone.0328407)
Supplement: Attachment — Submitted filename: Comments to the Author.pdf [file pone.0328407.s010.pdf]

General comments:

Thank you to the authors for submitting a manuscript for consideration for publication in PLOS One. Motivated to investigate how the neurocognitive processes underlie prosocial disobedience, the manuscript reports research using functional magnetic resonance imaging (fMRI) on civilians and military personnel. There are several concerns regarding the manuscript as outlined in the specific comments below:

Specific comments:

[Point 1] Abstract: Please spell fMRI in full when it is first time used and put the acronym in a bracket. The same goes for TPJ and dmPFC.

[Point 2] Abstract: Please highlight the significance or implication of the study at the end of the abstract.

[Point 3] Introduction: Please spell the following terms in full when it is first time used and put the acronyms in a bracket: fMRI, EEG, tDCS, ERP, NGO, SPM12, GLM. It is important to keep in mind that the readers of this journal come from diverse academic backgrounds and some may not be familiar with these terminologies. Moreover, it is a common practice in academic writing to spell any acronyms in full when they are first time introduced.

[Point 4] Introduction: Please provide the relevant reference to “literature” (line 48), “existing research” (line 49), and “several EEG and fMRI studies” (line 58). As a reader, I am interested to know the sources of the findings.

[Point 5] Introduction: Please replace “<u>a</u> fMRI study” with “<u>an</u> fMRI study” (line 71).

[Point 6] Introduction: Who were the “participants” in line 68? The same question goes to studies (4), (5), and (6). Were they civilians or military personnel? Please clarify.

[Point 7] Introduction: Why are the references “(Caspar, Rovai, Lo Bue, Cleeremans, 2024)”, “(Paus et al., 1997; Villena-González et al., 2018)”, “(Tricoche, Rovai, Caspar, 2024)” cited this way and not in numbering format? Please clarify.

[Point 8] Introduction: Please explain why the previous studies have not focused on disobedience. Could it be because that was not part of their objectives or disobedience was not an issue highlighted in the literature?

[Point 9] Introduction: Most of the studies highlighted in the introduction used EEG. However, in this study, the imaging modality of choice is MRI. Please explain the reasons for using fMRI, as opposed to EEG. What are the strengths (or limitations) of this technique?

[Point 10] Introduction: The novelty of this work is not clear. From the writing, it seems the uniqueness of the present work is that it only involved military populations (lines 136-137), which is also arguable considering there have been publications on these populations.

[Point 11] Introduction: Please state the significance of the work at the end of the introduction section. This would help readers see why was the study necessary and whether it contributes to our understanding of prosocial disobedience. The impact of the study should be general and specific.

[Point 12] Introduction: Overall, the introduction section has extensively discussed studies on disobedience. However, the introduction inadequately addresses the main problem that exists in the literature regarding disobedience. Though the authors did mention that “Understanding how military personnel transition between compliance and resistance, as well as how their daily environment impacts these decisions, is a significant societal and scientific issue that has not been approached in current literature” (lines 53-55), the authors did not explain further

what societal and scientific issues that may arise due to disobedience. The introduction section can be improved by highlighting reports on incidents that occur as a result of prosocial disobedience. Then, based on these reports, it would seem clearer why was the study necessary to be carried out.

[Point 13] Methodology: Please state the study design and the sampling method (both military and civilian populations).

[Point 14] Methodology: Please justify including 54 military officers and 56 civilians in the study by describing the method of sample size calculation. Additionally, please state the confidence level and statistical power of this sample size.

[Point 15] Methodology: Why was a participant excluded after finding out that he/she had a brain abnormality during the scanning session? The same goes for the other two participants who were excluded due to a technical problem. Were these part of the exclusion criteria? Speaking of which, please provide the study's inclusion and exclusion criteria.

[Point 16] Methodology: Did the civilians provide written informed consent before taking part in the study? This was not mentioned in the manuscript.

[Point 17] Methodology: Which group of "participants" (line 210) is this; civilian or military?

[Point 18] Methodology: Were the military agents trained individually or in a group?

[Point 19] Methodology: The following parameters need to be added for the fMRI sequence: field-of-view (FOV), acquisition order (e.g. descending, ascending, or interleaved), and acquisition time (i.e. the duration in which each EPI scan was acquired).

[Point 20] Methodology: Please explain why was the structural T1-weighted scan performed between the two functional scans. Normally, the structural scan is performed before the functional scans. How did the researchers plan the imaging sequence for the first functional run without an anatomical image of the participants' brains? This is indeed an interesting approach.

[Point 21] Methodology: How long was the fMRI session? Please clarify.

[Point 22] Methodology: How many functional volumes were acquired in total and how many were discarded (if any)? Please clarify.

[Point 23] Methodology: Please add a reference for SPM12 (line 327).

[Point 24] Methodology: Please explain the reason for using ROIs of a previous study (19) and not based on the brain areas that showed significant activation from the whole brain analysis.

[Point 25] Results: Did the authors perform a normality test to check the distribution of the data and ensure they met the assumptions of subsequent analyses (e.g. ANOVA)? If yes, please state the name of the normality test and report the results.

[Point 26] Results: Please provide the demographic data.

[Point 27] Discussion: While the discussion comprehensively discusses the results, it does not, however, highlight the implications of the findings. For instance, it is now clear that civilians and military personnel behave differently towards prosocial disobedience (which is already expected considering their different backgrounds), and that the neurocognitive processes underlying prosocial disobedience differ between them, but how these findings can be translated into practice needs to be further elucidated.

[END OF REVIEW]
