# Supplementary material for: Neuro-cognitive specificities in prosocial disobedience: A comparative fMRI study of civilian and military populations
Source: PLoS One. 2025 Jul 22;20(7):e0328407. doi: 10.1371/journal.pone.0328407 (PMC12282893; doi:10.1371/journal.pone.0328407)
Supplement: Attachment — Submitted filename: Tricoche-Rovai-LoBue&Caspar_Rebuttal Letter.docx [file pone.0328407.s012.docx]

We would like to warmly thank the editor and the two anonymous reviewers for their time in reading and commenting the manuscript. We have provided below a point-by-point response to the journal requirements and reviewers’ comments (text in blue) and all modifications in the manuscript and Supplementary Material are highlighted in blue.

**Journal Requirements:**

1. When submitting your revision, we need you to address these additional requirements.

Please ensure that your manuscript meets PLOS ONE's style requirements, including those for file naming. The PLOS ONE style templates can be found at

https://journals.plos.org/plosone/s/file?id=wjVg/PLOSOne_formatting_sample_main_body.pdf and

<https://journals.plos.org/plosone/s/file?id=ba62/PLOSOne_formatting_sample_title_authors_affiliations.pdf>

We adapted our manuscript in the required format.

2. Thank you for stating the following financial disclosure:

E.C: ERC Starting Grant DISOBEY (grant number: 101075690)

E.C: BOF Starting Grant from Ghent University (grant number: BOF/STA/202109/025)

Please state what role the funders took in the study.  If the funders had no role, please state: "The funders had no role in study design, data collection and analysis, decision to publish, or preparation of the manuscript."

If this statement is not correct you must amend it as needed.

Please include this amended Role of Funder statement in your cover letter; we will change the online submission form on your behalf.

Please see the amended Role of Funder Statement: *“The funders had no role in study design, data collection and analysis, decision to publish, or preparation of the manuscript”.*

3. Thank you for stating the following in the Acknowledgments Section of your manuscript:

The project was supported by an ERC Starting Grant DISOBEY (grant number: 101075690) and by a BOF Starting Grant from Ghent University (grant number: BOF/STA/202109/025) granted to Emilie A. Caspar. Views and opinions expressed are however those of the author(s) only and do not necessarily reflect those of the European Union or the European Research Council Executive Agency. Neither the European Union nor the granting authority can be held responsible for them.

We note that you have provided funding information that is not currently declared in your Funding Statement. However, funding information should not appear in the Acknowledgments section or other areas of your manuscript. We will only publish funding information present in the Funding Statement section of the online submission form.

Please remove any funding-related text from the manuscript and let us know how you would like to update your Funding Statement. Currently, your Funding Statement reads as follows:

E.C: ERC Starting Grant DISOBEY (grant number: 101075690)

E.C: BOF Starting Grant from Ghent University (grant number: BOF/STA/202109/025)

Please include your amended statements within your cover letter; we will change the online submission form on your behalf.

The Acknowledgment section was removed from the manuscript as it only contained the Funding Statement.

This is the Funding Statement (no change):

*“E.C: ERC Starting Grant DISOBEY (grant number: 101075690)*

*E.C: BOF Starting Grant from Ghent University (grant number: BOF/STA/202109/025)”*

4. Please amend either the abstract on the online submission form (via Edit Submission) or the abstract in the manuscript so that they are identical.

We corrected.

5. Please include captions for your Supporting Information files at the end of your manuscript, and update any in-text citations to match accordingly. Please see our Supporting Information guidelines for more information: http://journals.plos.org/plosone/s/supporting-information.

We adapted our Supplementary material following the guidelines and added the captions in the revised version of the manuscript (lines 1042 to 1111).

**Reviewers Comments to the Authors:**

**Reviewer #1:**

In their manuscript, „Neuro-cognitive specificities in prosocial disobedience, A comparative fMRI study of civilian and military populations”, Tricoche and colleagues compare different behavioral and neural correlates of (prosocial) disobedience. The find that civilians and military participants differ with respect to the stated reasons for disobedience as well as the neural regions most activated during the decision to disobey.

The research question of which aspects and potential determinants of (prosocial) disobedience may differentiate a civilian and a military population is interesting and the data thus of general interest to the community. I also very much welcome the data sharing and preregistration of the project, the hypotheses, and the analyses.

However, in its current form, the manuscript would benefit from a lot of (re-)focussing especially regarding the introduction and I think the authors should be more prudent when interpreting the fMRI results to avoid jumping to conclusions. That said, I think the points I raise can all be well addressed.

I hope my comments will help clarify, streamline, and improve the manuscript to ultimately move the manuscript towards publication.

Major:
1) The introduction is currently hard to follow and it would be very helpful to streamline the information provided towards the specific research questions. In detail, it may be helpful to focus on the 2-3 studies that most closely resemble the current study design and summarize findings from additional studies very briefly to support the fact that there is work out there showing a specific thing. E.g. study 32 seems to be especially relevant but is only mentioned quite late in the introduction. This will also be help to avoid the listing of too many brain regions that may or may not be more or less relevant to the actual research question.

Following the two reviewers comments we substantially revised the introduction of the manuscript by addressing their concerns. Please find the complete new version in the revised manuscript (lines 50 to 146).

2) The first page of the introduction needs more references. E.g. which studies have started investigating disobedience with fMRI, EEG, and tDCS (line 49), or which studies show that obedience may hold a different significance to university students vs. military personel (line 51), also line 59 (coercion effect), and 61 (sentence ending with “e.g. prosocial disobedience”). Please be mindful of supporting your claims by references directly where you make the claim.

We have added references associated to our statements: “*While this question has been explored in recent years through neuroimaging techniques such as functional Magnetic Resonance Imaging (fMRI), electroencephalography (EEG), and transcranial direct-current stimulation (tDCS) (1–3), the majority of the existing research have involved civilian participants—most often university students representing classic convenience samples, raising concerns about the generalizability of the findings (see for instance (1,2,4,5)”.* (lines 51 to 56).

For the ones associated to the coercion effect, we now have: *“Prior studies in civilian adult participants have shown that compliance with or resistance to orders to inflict pain to another person involves cognitive and affective processes such as empathy (2,6–9), guilt (2,7,8), mentalizing (2,6), cognitive conflict (2,10), and sense of agency (SoA) (2,6,8,9,11–13). These processes are engaged at different moments of the decision-making timeline (14): from the initial processing of an order given by the experimenter (i.e., pre-decision phase), to the act of deciding to obey or disobey (i.e., decision-making phase), to the evaluation of its consequences for the victim (i.e., post-decision phase). Notably, during post-decision, when witnessing the victim’s pain, reduced activity in empathy for pain and guilt-related brain networks was observed when participants complied with orders to cause pain compared to acting freely (7,9). This result may help explain how obedience reduces our natural aversion to inflicting harm on others. Moreover, during the decision-making phase, studies in civilian participants revealed at both behavioral and brain levels that following orders led to a reduction of cognitive conflict and diminished SoA, which may facilitate the adoption of a non-moral behavior (8–13,15,16). Interestingly, one previous work (13) showed similar neural patterns related to the SoA between civilians and military officers. However, these studies did not focus on disobedience per se in their research questions, primarily because adult participants almost never disobeyed orders in these experimental paradigms, making it impossible to compare the two types of decisions”* (lines 78 to 94).

3) The whole work would benefit from more theoretical and conceptual grounding. For example, what are the exact assumed mechanisms that are expected to differ between the two population? Why exactly would that be the expectations? What are the assumed differences between resisting to an order and disobedience as investigated here (cf. line 92)? This will also greatly help with the interpretation of the fMRI results, I think.

We have improved the last paragraph of the introduction to better develop our hypotheses and predictions: *“Almost no studies have compared civilian to military participants. However, since military officer cadets are more accustomed than civilians to receiving orders from authority figures, we hypothesized that they would show lower levels of disobedience. The reasoning behind their decisions might also differ due to their distinct educational environment and varying relationship with obedience. Moreover, two previous studies showed that SoA was the same among military officer cadets and civilians (8,13), and that it involved the same neural mechanisms (13). We could thus expect similar brain regions involved during the decision phase among the two populations. However, as disobedience is less endorsed in military officer cadets and could pose a greater psychological challenge in the context of their professional identity, the involvement of this neural activity could differ. For other processes in the pre-decision and post-decision phase (i.e., cognitive conflict, empathy, guilt), our hypotheses remain bidirectional, as no previous studies exist with our target population”* (lines 136 to 146).

4) Throughout the manuscript certain brain regions are labelled as e.g. “guilt-related” (ACC, TPJ, and basalganglia, line 75) or neural results from a previous study appear to be used to motivate behavioral predictions for the current one (line 146-149). I would strongly encourage the authors to be more mindful of the actual connection between activation in certain brain areas and behavior as especially regions such as the ACC and TPJ clearly are implicated in a variety of social as well as non-social cognitive processes. I also was surprised to read in lines 112/113 that left-lateralization “in response to immoral situations has been observed for the TPJ[…]” and one page later (line 128) "cathodal stimulation of the right TPJ showed an increased tendency to harm" an avatar. This looks like a contradiction to me. I am not opposed to lateralization per se, but in the present paradigm, I am not convinced it holds the key to a valuable understanding of the results.

As we substantially revised the Introduction section, the description of previous studies has been streamlined, which already addresses several of the reviewer’s concerns. The literature on prosocial disobedience is now presented as follows: *“The literature on prosocial disobedience remains relatively limited but reveals consistent engagement of social and moral cognition networks, pointing to the emotional and physiological cost of resisting orders (3,4). Specifically, a recent fMRI study with university students representing civilians found that during the decision-making period, up until the action, regions associated with moral judgment—specifically, the angular gyrus extending to the inferior occipital gyrus (AG/IOG), as well as the TPJ (17,18)—were negatively modulated in the disobedience condition compared to obedience (2). Increasing activity in this region has previously been linked to a reduced SoA (19), suggesting that a negative modulation of the AG during decision-making could help maintain a sense of responsibility, thereby enabling resistance to the experimenter’s order. Additionally, correlational analyses revealed a positive relationship between the rate of prosocial disobedience and the activity of medial regions when the decision to send a shock was made, particularly in the dorsomedial and ventromedial prefrontal cortex (dmPFC/vmPFC), the ACC, and the supplementary motor area (SMA) (2). These regions are extensively involved in the process of mentalizing, including the distinction between self and others, and are therefore crucial in complex social situations that require different perspectives to be taken into account (20–22). In the post-decision phase, Tricoche, Rovai and Caspar (2024) also found a positive correlation between the tendency to disobey and number of mentalizing regions over the fronto-parietal axis, more activated when the victim received a shock than a no shock. It included mPFC, SMA, superior parietal lobule (SPL), supramarginal gyrus (SMG), TPJ, AG, precuneus (Prec), PCC and anterior insula (AI). These findings suggested heightened sensitivity to the consequences of their decisions, ultimately helping civilians to disobey more by improving the victim’s perspective. Further support for the importance of mentalizing regions in prosocial disobedience comes from neurostimulation studies. For instance, cathodal stimulation of the right TPJ in Chinese adults, in a virtual replication of Milgram’s paradigm, led to shortened reaction times to obey to harm an avatar, suggesting that disrupting other’s perspective-taking processing can reduce resistance to immoral orders (1)”* (lines 95 to 119).

5) I was surprised to see that the main behavioral DV is a percentage of disobedience but does not seem to be relative to the relevant number of trials. This seems make the analysis less informative than it couled be imo (“As the number of instructions so give a shock was higher than the order to not give a shock, the main effect of instruction was expected”). You really want to avoid this. The percentage should be more interepretable relative to the number of trials in which the instructions was to shock vs. not too shock rather than relative to the whole 120 trials as it appears to be now. I was also wondering, why the authors did not opt for the analysis of trial-by-trial data using, e.g. logistic mixed-effects models. I think this would be very beneficial and also facilitate interpretation of the results.

We acknowledge the reviewer’s concern regarding how the disobedience percentage was calculated. In the current study, our main behavioral variable—the percentage of disobedience—was computed in the same way as in a previously published civilian dataset (Tricoche, Rovai & Caspar, 2024), which serves as the comparative baseline in this work. To ensure that our approach did not introduce interpretational bias, we recomputed disobedience percentages using only the relevant instruction trials (i.e., the 80 “send shock” trials where disobedience was possible). We found that the correlation between this refined measure and our original index (based on the full set of 120 trials) was virtually perfect (r ≈ 1). This confirms that both metrics capture the same behavioral variance, which makes sense given that this is a ratio. Given this result, and for the sake of methodological consistency and comparability with the previously published civilian dataset using the original approach, we prefer to maintain the initial measurement in the main analysis.

Regarding the use of logistic mixed-effects models, we agree that a trial-level analysis would offer a valuable complementary perspective. However, our analysis choice was guided by methodological considerations inherent to our within-between-subject design. Specifically, the behavioral data in our task are binomial by nature and result in relatively unbalanced response distributions for certain conditions (e.g., antisocial disobedience), which creates convergence issues in generalized linear mixed-effects models (GLMMs), particularly with random slopes. Particularly, the number of parameters (induced by the design’s complexity) and the sparsity of certain response types per subject further complicate the estimation of reliable GLMMs without overfitting or excluding a significant portion of participants. For these reasons, we opted for aggregated analyses that remain well-aligned with our pre-registered hypotheses (https://osf.io/3mt4d) and ensure robust, interpretable results. That said, we acknowledge the potential value of mixed-effects modeling in future work, particularly in studies designed with higher trial counts by decision type or more balanced response distributions to support such analyses.

6) A further boost of explanatory evidence could be gained from relating the stated reasons for disobeying to the brain activation or contrasts of interest. This way, the results could paint a more comprehensive picture of which factors do or don’t influence differential neural correlates of disobedience in civilian and military participants.

We agree the reviewer that this supplementary analysis could be of interest. However, as it is an exploratory analysis not taking part of our initial hypotheses, we decided to focus on the relationship between activity of ROIs which showed a significant difference between the two populations (i.e., bilateral AI, right TPJ, Prec-PCC), and on the reasons of disobedience that reached significance (i.e., morality, sensitivity, education). The regressions related to the other reasons of disobedience (i.e., shock quantity, money, entertainment, rejection of authority, experimental, fear of judgment, and country’s history criterions) are reported in Supplementary Information.

In the Method section we added: *“As an exploratory analysis, we also conducted segmented regressions between activity of ROIs showing significant difference between the two populations and the disobedience’s criteria (Model 1: ROI activity ~ Criterion x Population; Model 2: Criterion ~ ROI activity x Population)”* (lines 43	2 to 435).

In the Result section its now written: *“As an exploratory analysis, we conducted segmented regressions between the activity of these ROIs and disobedience-related criteria. We primarily focused on the three criteria that were significantly correlated with %Pro_disob (sensitivity, morality, and education). Sensitivity and education criteria did not show significant relationships with brain activity in any ROI, nor did they show interactions with the population factor. However, a stronger negative relationship was observed between the morality criterion and activity in the left AI in military officer cadets compared to civilians. This suggests that the increased recruitment of the left AI during disobedience among military officer cadets, relative to civilians, was not primarily explained by higher self-reported morality criterion. Full results for the other disobedience-related criteria are provided in S7 file. Interestingly, these analyses revealed stronger associations between right TPJ and Prec/PCC activity and both the "country’s history" and "rejection of authority" criteria in military officer cadets compared to civilians. For the rejection of authority criterion, a significant relationship was also observed with left AI activity. Taken together, these findings suggest that in military officer cadets, the greater recruitment of these brain regions during decision-making, associated to higher prosocial disobedience, is also linked to higher subjective importance attributed to rejecting authority and acknowledging the historical role of their country”* (lines 698 to 713).

We addressed this supplementary result in the Discussion section: *“Interestingly, we also found a positive relationship between the activity of these brain regions and the subjective importance attributed to rejection of authority and the historical role of the country. This suggests that, for military officer cadets, the successful enactment of prosocial disobedience may be supported not only by cognitive-emotional mechanisms, but also by broader socio-cultural values that reinforce critical evaluation of authority”* (lines 803 to 807).

Supplementary Information numbers were adapted. We added a S7 file:

***S7 File. Segmented regression model analyses between disobedience’s criteria, significant ROIs, and population.***

*Segmented regression model analysis between the ROIs (i.e., bilateral AI, right TPJ and Prec/PCC) and disobedience’s criteria (i.e., shock quantity, money, entertainment, rejection of authority, experimental, fear of judgment, and country’s history criteria), taking the Population factor (Civilians, Military) into account. Both models are considered: ROI’s activity ~ criterion x Population (model 1) and criterion ~ ROI’s activity x Population (model 2).*

*Shock quantity, fear of judgment, entertainment and money criteria did not show significant relationship with ROIs activities, nor interaction with Population (all p’s>0.05).*

*Country’s history criterion showed a stronger positive relationship with right TPJ and Prec/PCC activities in military officer cadets than civilians, particularly for model 1 (Model 1: p=0.01 for right TPJ and p=0.009 for Prec/PCC; Model 2: p=0.08 for right TPJ, p=0.06 for Prec/PCC).*

*Rejection of authority criterion showed a stronger positive relationship with left AI, right TPJ and Prec/PCC activities in military officer cadets than civilians (Model 1: p=0.01 for left AI, p=0.01 for right TPJ, p=0.005 for Prec/PCC; Model 2: p=0.02 for left AI, p=0.09 for right TPJ, p=0.04 for Prec/PCC).*

*Finally, for the experimental criterion only a significant main effect emerged showing that left AI activity negatively influenced this criterion (Model 1: p=0.04), with no interaction with the Population (all others p’s>0.08).*

Minor:
1) Please specify the shape and extent of the imaging ROIs (line 383). Did you use spheres or atlas-based ROIs, or something completely different? Helpful to know here.

We think this information was already given in the initial version of the manuscript: “*In each ROI (built as the intersection of 10mm radius spheres centered on the local maximum of each cluster), the activity was averaged across all voxels. The mean beta values were extracted from both the [“Send a shock”/Obedience > “Send a shock”/Disobedience] (i.e., same order but different choices) and the [“Send a shock”/Obedience > “Do not send a shock”/Obedience] (i.e., different orders but similar choices) contrasts for each ROI”* (lines 422 to 426)*.*

2) It would be helpful to include a clearer explanation for the SoA measure and what exactly it means. I appreciate that the authors always included the information about what high and low values mean, respectively but I think it will be helpful to expand on this. I also gathered from section 3.1.2 that the SoA was lower for disobedience than for obedience decisions. This seems quite counter-intuitive, right? Would be helpful to discuss this.

In the introduction part we added the definition of SoA: *“[…] and sense of agency (SoA ; defined as the subjective experience of controlling one’s actions) (2,6,8,9,11–13)”* (lines 80-81).

In the initial version of the manuscript, in the Method section, we first defined the temporal binding task measuring SoA: *“Military officer cadets had 6 seconds to estimate the action-outcome delay corresponding to the interval between the keypress and the tone (12), using a temporal binding scale from 0 to 1,000ms. The temporal binding task is an implicit method to measure SoA based on time perception (27,28). More precisely, previous studies showed that performing voluntary actions led to a compression of time between the action and the outcome, compared to when the action was performed involuntarily (i.e., temporal binding effect) (29,30)”* (lines 249 to 254).

The following lines specifically explain the way we measured SoA (with two complementary measures): “*Implicit Sense of Agency (SoA) was targeted by measuring the temporal binding score, similarly to previous studies (7,8,11). During the Agency run participants were asked to estimate the duration (0 to 1,000ms) between their keypress (action) and the tone associated to the shock/no shock (outcome). Interval estimates, IE, were transformed into z-scores: z-score =* $\frac{{IE}_{i}-mean IEs}{sd IEs}$*, where i corresponds to one trial. We also calculated the mean difference between the IE and the actual delay (error-score), as a second indicator of temporal binding. Then, higher IEs (i.e., positive z-score or positive error-score) are associated with a lower SoA; and reciprocally*” (lines 293 to 299).

We agree with the reviewer that a lower SoA during disobedience compared to obedience may seem somewhat surprising—although not entirely counterintuitive given the military population involved in the present study. For military personnel, disobedience may be perceived as a less natural decision than obedience, potentially resulting in a lower initial sense of agency when disobeying compared to following orders. However, our correlation analyses show that the more participants disobey, the higher their reported SoA. This suggests that, through other mechanisms and cognitive processes, they may be able to override this natural tendency and experience a stronger sense of engagement and ownership over their decision to disobey. In the discussion part we rephrased and wrote: *“Moreover, our behavioral results indicated a lower SoA when military officer cadets disobeyed, compared to when they obeyed — suggesting a reduced sense of authorship over their actions when defying instructions from an authority figure, as opposed to following orders. This may be because, for military personnel, resisting orders is not a conditioned behavior due to their training. However, correlational analyses showed that the more military officer cadets disobeyed, the more their SoA increased. This result, combined with the observed neural activities, suggests that military officer cadets may had a lower natural tendency to disobey; to do so, they seemed to engage emotional regions and focused on self-reflection, thereby enhancing their sense of responsibility and agency, which ultimately enabled disobedience”* (lines 807 to 815).

3) I typically don’t use segmented regression and was not familiar with the concept until reviewing this paper. Keeping in mind that other readers (I also asked other methodologically interested colleagues) may also not be familiar with it, it may be helpful to include a one sentence explainer what it is and how the interpretation of the results relate to other types of regression (e.g. in segmented regression, the population effect appears to be a main effect whereas in linear regression, the same effect would have been an interaction effect – if understood correctly).

We now better justified and explained this analysis in the Method section: *”To investigate if the relation between the %Pro_disob and the behavioral (scale scores) or subjective measures (debriefing sub-scales) was modulated by the Population (Military, Civilians), we adjusted a segmented linear regression model to the data for each run (‘segmented’ package on R). This approach was chosen because it allows for the detection of nonlinearities or structural changes in the relationship between variables (%Pro_disob and Population) —particularly useful when interactions with categorical groups (such as Population) may result in different patterns. Indeed, unlike standard linear regression, which assumes a single, constant slope across the entire range of the independent variable, segmented regression allows the model to estimate different slopes, informing when a relationship shifts at a certain threshold, depending on the population. The relation was investigated in the two directions leading to two models: 1) Measure ~ %Pro_disob x Population; 2) %Pro_disob ~ Measure x Population. This bidirectional modeling strategy enabled us to explore whether disobedience rates predicted variations in behavioral or subjective responses and conversely, whether individual differences influenced disobedience rates, across the two populations”* (lines 319 to 332).

4) I recommend having a native speaker proof-read the manuscript. I spotted a few errors that could be weeded out to further improve readability of a revised version.

The manuscript has been revised accordingly to improve clarity and readability.

**Reviewer #2:**

Dear Authors,

Thank you to the authors for submitting a manuscript for consideration for publication in PLOS One. Motivated to investigate how the neurocognitive processes underlie prosocial disobedience, the manuscript reports research using functional magnetic resonance imaging (fMRI) on civilians and military personnel.

General comments:

Thank you to the authors for submitting a manuscript for consideration for publication in PLOS One. Motivated to investigate how the neurocognitive processes underlie prosocial disobedience, the manuscript reports research using functional magnetic resonance imaging (fMRI) on civilians and military personnel. There are several concerns regarding the manuscript as outlined in the specific comments below:

Specific comments:

[Point 1] Abstract: Please spell fMRI in full when it is first time used and put the acronym in a bracket. The same goes for TPJ and dmPFC.

We corrected the abstract.

[Point 2] Abstract: Please highlight the significance or implication of the study at the end of the abstract.

In the abstract we have now written: *“These findings could have implications for ethics training in military and institutional contexts, and offer insights into how obedience and resistance are cognitively and emotionally constructed across different social roles and institutional environments”* (lines 39 to 41).

[Point 3] Introduction: Please spell the following terms in full when it is first time used and put the acronyms in a bracket: fMRI, EEG, tDCS, ERP, NGO, SPM12, GLM. It is important to keep in mind that the readers of this journal come from diverse academic backgrounds and some may not be familiar with these terminologies. Moreover, it is a common practice in academic writing to spell any acronyms in full when they are first time introduced.

We corrected by adding the associated terms at first use.

[Point 4] Introduction: Please provide the relevant reference to “literature” (line 48), “existing research” (line 49), and “several EEG and fMRI studies” (line 58). As a reader, I am interested to know the sources of the findings.

We added references associated to the “literature” and “existing research” terms: “*While this question has been explored in recent years through neuroimaging techniques such as functional Magnetic Resonance Imaging (fMRI), electroencephalography (EEG), and transcranial direct-current stimulation (tDCS) (1–3), the majority of the existing research have involved civilian participants—most often university students representing classic convenience samples, raising concerns about the generalizability of the findings (see for instance (1,2,4,5)”.* (lines 51 to 56).

For the ones associated to the coercion effect, we know have: *“Prior studies in civilian adult participants have shown that compliance with or resistance to orders to inflict pain to another person involves cognitive and affective processes such as empathy (2,6–9), guilt (2,7,8), mentalizing (2,6), cognitive conflict (2,10), and sense of agency (SoA) (2,6,8,9,11–13). These processes are engaged at different moments of the decision-making timeline (14): from the initial processing of an order given by the experimenter (i.e., pre-decision phase), to the act of deciding to obey or disobey (i.e., decision-making phase), to the evaluation of its consequences for the victim (i.e., post-decision phase). Notably, during post-decision, when witnessing the victim’s pain, reduced activity in empathy for pain and guilt-related brain networks was observed when participants complied with orders to cause pain compared to acting freely (7,9). This result may help explain how obedience reduces our natural aversion to inflicting harm on others. Moreover, during the decision-making phase, studies in civilian participants revealed at both behavioral and brain levels that following orders led to a reduction of cognitive conflict and diminished SoA, which may facilitate the adoption of a non-moral behavior (8–13,15,16). Interestingly, one previous work (13) showed similar neural patterns related to the SoA between civilians and military officers. However, these studies did not focus on disobedience per se in their research questions, primarily because adult participants almost never disobeyed orders in these experimental paradigms, making it impossible to compare the two types of decisions”* (lines 78 to 94).

[Point 5] Introduction: Please replace “a fMRI study” with “an fMRI study” (line 71).

We have corrected.

[Point 6] Introduction: Who were the “participants” in line 68? The same question goes to studies (4), (5), and (6). Were they civilians or military personnel? Please clarify.

We clarified at several points in the introduction that the studies involved adult participants and distinguished between civilian and military populations.

[Point 7] Introduction: Why are the references “(Caspar, Rovai, Lo Bue, Cleeremans, 2024)”, “(Paus et al., 1997; Villena-González et al., 2018)”, “(Tricoche, Rovai, Caspar, 2024)” cited this way and not in numbering format? Please clarify.

We thank the reviewer for these mistakes. We corrected.

[Point 8] Introduction: Please explain why the previous studies have not focused on disobedience. Could it be because that was not part of their objectives or disobedience was not an issue highlighted in the literature?

Previous studies initially focused on the process of obedience, using experimental paradigms specifically designed to address that research question. However, within these paradigms, participants typically did not disobey, making it impossible to include disobedience as a factor in the analyses. By turning their attention to the mechanisms underlying prosocial disobedience, researchers first had to develop a paradigm in which both obedience and disobedience decisions occurred frequently enough to allow for meaningful comparison. As a result, studies explicitly investigating disobedience remain scarce, and only a limited number of experimental paradigms currently exist.

In the Introduction part we stated: *“However, these studies did not focus on disobedience per se in their research questions, primarily because adult participants almost never disobeyed orders in these experimental paradigms, making it impossible to compare the two types of decisions”* (lines 92 to 94).

[Point 9] Introduction: Most of the studies highlighted in the introduction used EEG. However, in this study, the imaging modality of choice is MRI. Please explain the reasons for using fMRI, as opposed to EEG. What are the strengths (or limitations) of this technique?

Both in studies on obedience and disobedience, the use of EEG and fMRI has been relatively balanced. For instance, studies such as *Caspar et al., 2020; Caspar et al., Cerebral Cortex, 2025; Caspar et al., PlosOne, 2021* in obedience or *Tricoche, Rovai & Caspar, Imaging Neurosci., 2024* in disobedience, have employed fMRI, while others like *Caspar & Pech, Soc. Neurosci. 2024; Caspar et al., Current Biol. 2016* in obedience or *Caspar, Gishoma et al., Sci. Rep., 2022* in disobedience, have used EEG; and one even merged both techniques: *Caspar et al, eNeuro, 2021*. The combination of these two imaging modalities has provided complementary insights—EEG offering precise temporal markers of neural activity, and fMRI enabling the identification of broader brain networks involved in these processes.

In the present study, our goal was to compare civilians and military personnel (matched by age and region of residence). To ensure methodological consistency and allow for meaningful comparisons between the two populations, we adopted the same fMRI-based protocol as in Tricoche, Rovai & Caspar (2024). In the manuscript we added: *“Their behavioral and brain-level results were compared with a previously published civilian dataset (2) using the same paradigm and fMRI scanner”* (lines 134 to 135).

[Point 10] Introduction: The novelty of this work is not clear. From the writing, it seems the uniqueness of the present work is that it only involved military populations (lines 136-137), which is also arguable considering there have been publications on these populations.

Following the two reviewers comments we substantially revised the introduction of the manuscript by addressing their concerns. Please find the complete new version in the revised manuscript (lines 50 to 146).

Particularly we are now stating:

*“However, investigating these questions within military populations is a crucial societal and scientific endeavor, as both historical and contemporary events demonstrate that obedience to authority can lead to tragic outcomes. For instance, the Brereton Report (2020) investigated war crimes committed by Australian soldiers, and reported incidents in which junior soldiers were ordered to execute prisoners of war as part of a "blooding" ritual (https://www.defence.gov.au/about/reviews-inquiries/afghanistan-inquiry, accessed on April 2025). This example highlights the profound human consequences of uncritical compliance and underscores the urgent need to understand how and when individuals—particularly those in military contexts—choose to resist immoral orders. Yet, military personnel, who are regularly required to carry out commands, must also be capable of evaluating the legitimacy of those orders and disobeying when necessary, in accordance with international humanitarian law (United Nations, 1950). Thus, resistance to authority is not only a psychological phenomenon but also a matter of critical societal importance—especially in contexts where following orders is routine”* (lines 59 to 70)*.*

*“Understanding how military personnel transition between compliance and resistance, as well as how their daily environment impacts these decisions, has not been approached in current literature. The present pre-registered study addresses this gap by investigating the neuro-cognitive mechanisms involved in resistance to immoral orders (i.e., prosocial disobedience) among military officer cadets and comparing them to civilians. Our goal is twofold: (1) to identify whether the same brain mechanisms observed in civilian populations are recruited when military participants choose to resist harmful orders, and (2) to determine whether the degree or nature of this recruitment differs across populations”* (lines 71 to 77)*.*

“*Despite these initial neural findings, the literature on prosocial disobedience remains limited and largely confined to civilian samples. The present study aims to expand this work by examining a group for whom obedience and disobedience are not only routine but institutionally prescribed — namely, military personnel. It specifically focused on prosocial disobedience, defined here as the refusal to comply with orders to inflict harm on another person. Indeed, it is important to distinguish between general disobedience and resistance to immoral orders specifically aimed at protecting others— as resistance rooted in moral concern could engage different mechanisms than disobedience driven by self-interest or norm compliance. Military officer cadets represent a particularly relevant population, as they are both trained to follow orders and educated in an ethical framework”* (lines 120 to 128).

In response to the reviewer’s comment, we would like to emphasize that, to the best of our knowledge, no study has yet investigated the neuro-cognitive processes underlying prosocial disobedience in military populations, that are highly relevant for these types of decisions. Additionally, this work provides a direct comparison between civilians and military officer cadets—a comparison that remains rare, even in the broader literature on obedience. There are only two existing studies with military population on related topics, but they did not target disobedience.

[Point 11] Introduction: Please state the significance of the work at the end of the introduction section. This would help readers see why was the study necessary and whether it contributes to our understanding of prosocial disobedience. The impact of the study should be general and specific.

Please, see our response in **Point 10**.

[Point 12] Introduction: Overall, the introduction section has extensively discussed studies on disobedience. However, the introduction inadequately addresses the main problem that exists in the literature regarding disobedience. Though the authors did mention that “Understanding how military personnel transition between compliance and resistance, as well as how their daily environment impacts these decisions, is a significant societal and scientific issue that has not been approached in current literature” (lines 53-55), the authors did not explain further what societal and scientific issues that may arise due to disobedience. The introduction section can be improved by highlighting reports on incidents that occur as a result of prosocial disobedience. Then, based on these reports, it would seem clearer why was the study necessary to be carried out.

Please, see our response in **Point 10**.

[Point 13] Methodology: Please state the study design and the sampling method (both military and civilian populations).

We did not fully understand what information was missing for the reviewer.

In the initial version of the manuscript, we already indicated 1) the sample size for each population, 2) the gender and age composition of each population, 3) the inclusion criteria, 4) all the detailed statistics informing about the study design, 5) a description of one trial as well as the difference between the two runs, and 6) the study design and procedure.

We added several details and clarified sentences:

1) *“This experimental study was preregistered on OSF (*[*https://osf.io/3mt4d*](https://osf.io/3mt4d)*)”* (line 149).
2) *“Fifty-four military officer cadets (3rd and 4th years students) were contacted and recruited through the Royal Military Academy of Belgium (22 identified as females, age range: 19-29 years, mean: 21.6+/-1.8 years). All participants agreed to take part in the study between February 8, 2023, and April 23, 2024, and completed the fMRI session in which they played the role of an agent. Their results were compared with an equivalent sample of 56 civilian participants living in Belgium (33 identified as females, age range: 19-36 years, mean: 23.7+/-4.2 years), who were recruited via social medias and previously analyzed (inclusion period: December 20, 2022 – May 15, 2023; under the same ethic approval), as reported in Tricoche, Rovai and Caspar, 2024 (2)”* (lines 157 to 164).
3) *“Participants had to meet the following inclusion criteria: being French, Dutch or English speakers; having normal or corrected vision; having no MRI contra-indication; and having no known neurological or psychological disorder”* (lines 164 to 166).
4) *“Military officer cadets completed the Agency and Empathy runs in a counterbalanced order, in a single session”* (lines 244 to 245).

[Point 14] Methodology: Please justify including 54 military officers and 56 civilians in the study by describing the method of sample size calculation. Additionally, please state the confidence level and statistical power of this sample size.

We apologize to the reviewer, as the power analysis had indeed been conducted for the pre-registration, but we mistakenly believed it had been included in the manuscript. This has now been corrected: *“Using GPower and R, a priori computation of sample size indicated that we had to recruit a total sample of 1) 26 participants with ANOVAs, 2) 75 participants with correlations, and 3) at least 80 participants with segmented regressions; to achieve a power of .85, with a medium effect size (f=0.25, r=0.3, f²=0.15) and a Type I error of 5%. To prevent unexpected data loss, we increased the sample size to at least 50 participants per group (i.e., a total of N ≥ 100) to ensure sufficient power for all our analyses”* (lines 152 to 157).

[Point 15] Methodology: Why was a participant excluded after finding out that he/she had a brain abnormality during the scanning session? The same goes for the other two participants who were excluded due to a technical problem. Were these part of the exclusion criteria? Speaking of which, please provide the study’s inclusion and exclusion criteria.

The first participant was excluded after the MRI scan revealed a significant anatomical abnormality in the left fronto-parietal region. Although this specific anomaly was not known prior to participation, it directly affected brain structure in regions central to our analysis, and therefore would have compromised data interpretation and comparability across participants. While this was not explicitly listed in the initial exclusion criteria, such structural abnormalities fall under the broader principle of excluding any condition that could bias neural measurements or limit the validity of the group-level analysis.

In the manuscript we detailed: *“However, one military officer cadet was discarded from the fMRI analyses after discovering a significant anatomical abnormality during the scanning session that could compromise interpretation of imaging results and comparability across participants”* (lines 166 to 169).

For the two other participants, this was not a formal exclusion criterion. Rather, their data could not be recorded due to a technical issue during the MRI acquisition, meaning there were simply no data available to include or exclude from the analysis. In the manuscript we specified: *“Two other participants were discarded from the fMRI analyses due to a technical problem during the MRI-data acquisition, which resulted in a complete absence of usable MRI data”* (lines 169 to 171).

Please see **Point 13** for the other inclusion criteria.

[Point 16] Methodology: Did the civilians provide written informed consent before taking part in the study? This was not mentioned in the manuscript.

**Yes, they did.** This information was already provided in Tricoche, Rovai, & Caspar (2024). In the manuscript we indicated that *“All participants signed a written informed consent […]”*. However, we acknowledge that it should have been better clarified. This detail has now been added to the manuscript: *“All participants (both civilians and military officer cadets) signed a written informed consent, and received a financial compensation between €30 and €40”* (lines 180 to 182).

[Point 17] Methodology: Which group of “participants” (line 210) is this; civilian or military?

For the sake of clarity throughout the manuscript, we have standardized the terminology referring to the military population by consistently using the term “military officer cadets”.

We have also added a sentence at the beginning of the Methods section to clearly state that the methodology described in the present manuscript is identical to that developed in Tricoche, Rovai, and Caspar (2024): *”The following methodology was identical for civilian participants, as detailed in Tricoche, Rovai & Caspar, 2024 (2)”* (lines 188 to 189).

[Point 18] Methodology: Were the military agents trained individually or in a group?

All participants, including both civilians and military officer cadets, were trained and pass the study individually, as MRI scans are individual (*“Military officer cadets came at the MRI scanner alone […]”* (line 190)). We specified: *“After signing the consent forms, military officer cadets were individually trained for the two runs (Agency and Empathy) to ensure they understood the tasks”* (lines 209 to 210).

[Point 19] Methodology: The following parameters need to be added for the fMRI sequence: field-of-view (FOV), acquisition order (e.g. descending, ascending, or interleaved), and acquisition time (i.e. the duration in which each EPI scan was acquired).

These parameters were added: *“Two runs, one for Agency and one for Empathy, of functional images were recorded in a single-shot echo planar imaging (EPI) sequence with the following parameters: matrix = 96x96; field-of-view (FOV) = 288 × 288 mm; number of slices = 45; TR = 2.2 seconds; TE = 20ms; flip angle = 90°; voxel size = 3x3x3mm³; slice thickness = 3mm; acquisition order = interleaved (bottom-up)). The Agency functional run lasted approximately 30 to 35 minutes, and the Empathy run approximately 20 to 25 minutes, depending on the number of volumes acquired per participant. The time of the Agency Run was longer as participants also had to complete a time estimation task as a proxy for SoA”* (lines 341 to 348).

[Point 20] Methodology: Please explain why was the structural T1-weighted scan performed between the two functional scans. Normally, the structural scan is performed before the functional scans. How did the researchers plan the imaging sequence for the first functional run without an anatomical image of the participants’ brains? This is indeed an interesting approach.

It appears there may be a confusion between the structural T1-weighted anatomical scan and the initial localizer scan typically acquired at the beginning of the scanning session. In our protocol, a localizer scan was indeed performed prior to any functional scans, as is standard practice. This allowed for the correct positioning and planning of the imaging volumes for the first functional run. The high-resolution structural T1-weighted image, however, was acquired between the two functional runs, in order to optimize session timing and minimize participant fatigue during task-related scans.

[Point 21] Methodology: How long was the fMRI session? Please clarify.

Participants remained in the scanner for approximately one and a half hours, including scanner calibration, the initial localizer scan, the two functional runs, the T1-weighted structural scan, and the resting-state acquisition.

We specified in the manuscript: *“Overall, the MRI session lasted approximately one and a half hours per participant”* (lines 352 to 353).

[Point 22] Methodology: How many functional volumes were acquired in total and how many were discarded (if any)? Please clarify.

The total number of functional volumes varied across participants, as it depended on individual response times during the task; therefore, it was not fixed. At the beginning of each functional run, four dummy scans were acquired to allow for magnetic field stabilization. No additional volumes were systematically discarded during preprocessing.

In the manuscript we specified: *“At the beginning of each functional run, four dummy scans were acquired to allow for magnetic field stabilization, and were automatically discarded by the scanner”* (lines 351 to 352).

[Point 23] Methodology: Please add a reference for SPM12 (line 327).

We added the following reference: <https://www.fil.ion.ucl.ac.uk/spm/software/spm12/>, accessed on April 2025.

[Point 24] Methodology: Please explain the reason for using ROIs of a previous study (19) and not based on the brain areas that showed significant activation from the whole brain analysis.

We used the same ROIs as those defined in Tricoche, Rovai & Caspar (2024) to ensure direct comparability between the two populations (military officer cadets and civilians) *> consistency in methodology*. The goal of this study was not to re-identify activation patterns from scratch, but rather to examine whether the same brain regions previously implicated in prosocial disobedience in civilians would show similar or different patterns of activation in military officer cadets.

[Point 25] Results: Did the authors perform a normality test to check the distribution of the data and ensure they met the assumptions of subsequent analyses (e.g. ANOVA)? If yes, please state the name of the normality test and report the results.

In the manuscript we added: *“The assumptions of normality were assessed through visual inspection of the residuals using histograms and Q–Q plots. The distributions appeared reasonably symmetric and did not show substantial deviations from normality, excepted for the pain score. However, ANOVA was retained for this measure due to the large sample size and its robustness under such conditions (31)”* (lines 307 to 310).

[Point 26] Results: Please provide the demographic data.

We added the demographic information for civilians (initially described in Tricoche, Rovai & Caspar, 2024): *“Fifty-four military officer cadets (3rd and 4th years students) were contacted and recruited through the Royal Military Academy of Belgium (22 identified as females, age range: 19-29 years, mean: 21.6+/-1.8 years). All participants agreed to take part in the study between February 8, 2023, and April 23, 2024, and completed the fMRI session in which they played the role of an agent. Their results were compared with an equivalent sample of 56 civilian participants living in Belgium (33 identified as females, range: 19-36 years, mean: 23.7+/-4.2 years), […]”* (lines 157 to 162).

For ethical reasons, and because it was not central to our research question, we did not collect additional demographic information.

[Point 27] Discussion: While the discussion comprehensively discusses the results, it does not, however, highlight the implications of the findings. For instance, it is now clear that civilians and military personnel behave differently towards prosocial disobedience (which is already expected considering their different backgrounds), and that the neurocognitive processes underlying prosocial disobedience differ between them, but how these findings can be translated into practice needs to be further elucidated.

We improved our conclusion paragraph to better highlight implications of our study and associated findings: *“Overall, our results suggest that one's environment can influence moral decisions and responses to orders from an authority figure. While we observed that the engaged processes are consistent across civilians and military officer cadets, the degree of engagement varied, with observed neural differences indicating distinct “strategies” for disobedience between military officer cadets and civilian participants. Notably, we observed that military officer cadets had a lower sense of agency when they disobeyed orders, even when they did so prosocially, compared to civilians. While previous studies tend to suggest that positive outcomes enhance the sense of agency (51,52), the reduction of SoA in military officer cadets for prosocial actions may suggest that disobeying is a more complex decision for them, for which they do not take full authorship, at least as measured with implicit measures. We also observed that military officer cadets relied more than civilians on emotional brain regions associated with the decision and post-decision phases, perhaps as a compensatory mechanism for the lower SoA. Additional studies are necessary to better understand how the processes involved in the different decision phases interact with each other and how they can compensate for each other’s involvement to achieve disobedience. These findings have practical implications, particularly in military and institutional contexts, including training and ethics education. While military personnel are taught that they are legally required to refuse unlawful orders under international humanitarian law (United Nations, 1950), little attention is typically given to the psychological and neurocognitive mechanisms that make such disobedience difficult to enact. The present study suggests that prosocial disobedience is not only a legal or moral obligation, but also a cognitively and emotionally demanding act, particularly for individuals embedded in hierarchical structures. Integrating our insights into training programs could be valuable: for example, by combining legal instruction with practical exercises that enhance soldiers’ awareness of the psychological and neural processes involved in disobedience, including how obedience can become a conditioned response, and how emotional and cognitive strategies may be recruited to overcome it. By highlighting these mechanisms, training could help normalize disobedience in situations where moral and law require it—not as defiance, but as a deliberate and reasoned act of ethical responsibility. Future work could explore how such training might increase the likelihood of prosocial disobedience and support a more balanced sense of agency among military personnel when they face ethically challenging decisions”* (lines 876 to 902).
