# Supplementary material for: Neuro-cognitive specificities in prosocial disobedience: A comparative fMRI study of civilian and military populations
Source: PLoS One. 2025 Jul 22;20(7):e0328407. doi: 10.1371/journal.pone.0328407 (PMC12282893; doi:10.1371/journal.pone.0328407)
Supplement: Attachment — Submitted filename: Tricoche-Rovai-LoBue&Caspar_Rebuttal_Letter_auresp_2.docx [file pone.0328407.s013.docx]

1) I was not able to locate data and code following the link provided in the manuscript. Under the Files tab in the OSF project, I could only see the manusciprt pdf file and the supplement pdf file. From what I gather, data (and possibly code) should be available as e.g., csv or txt file. In the case of imaging data, as .nii probably. Thus, it would be helpful to point the reader to where the data is at in the data availability link. If it was hidden somewhere in the project, I apologize, but would still recommend to make them easy to find.

We apologize for this issue. Data are indeed available at the following link: <https://osf.io/xcgak/>

We corrected in the manuscript.

2) Regarding my previous point on using the 80 trials of instructed shock in place of the 120 trials that cover all trials, I was surprised to see that the authors presented a correlation between those two options. Mathematically, any difference from a correlation unequal to 1 would be a results of rounding errors and is beside the point I tried to make. The key point I did aim to make was that the way the ANOVA is set up does not reflect the way the dependent variable is chosen. That is, if you include the factor of instruction (to shock vs. no to shock), the dependent variable should be instruction -specific. I think it would thus be helpful to include this analysis for both samples - even if only in the supplement - to demonstrate that the effects hold. An additional benefit would be that you would actually be able to interpret an interaction effect of decision X instruction. I do appreciate that it won't be necessary to rerun all analysis with this new %pro_disob.

We conducted the ANOVA using this revised calculation of percentage choice, as suggested by the reviewer. The results are provided in S2 File and are comparable to those obtained in the initial ANOVA.
We have added the following to the main text: *“To ensure the robustness of our findings, we also computed the percentage of choices relative to the number of trials corresponding to each instruction (“Send a shock” = 80 trials; “Do not send a shock” = 40 trials). The results, reported in S2 File, were comparable to those presented here”* (lines 467 to 470).

In S2 we now have: “*The four-way ANOVA led to the same significant effects as the one obtained for the initial percentage calculation. It revealed a main effect of Instruction (F(1,651)=16.68, p<0.001,* $\eta_{p}$*²=0.02,* ${BF}_{incl}$*>100 in favor of H1), a main effect of Choice (F(1,651)=389.94, p<0.001,* $\eta_{p}$*²=0.37,* ${BF}_{incl}$*>100 in favor of H1), as well as a significant interaction between these two factors (F(1,651)=111.52, p<0.001,* $\eta_{p}$*²=0.15,* ${BF}_{incl}$*>100 in favor of H1). The main effect of Population (F(1,97)=30.67, p<0.001,* $\eta_{p}$*²=0.24,* ${BF}_{incl}$*>100 in favor of H1) and the Choice x Population interaction were also significant (F(1,651)=14.94, p<0.001,* $\eta_{p}$*²=0.02,* ${BF}_{incl}$*>100 in favor of H1). We did not find a significant main effect of Run nor interactions with the other factors (all ps>0.40, all* ${BF}_{incl}$*<3 in favor of H0). The only difference with the main analysis was a significant Instruction x Choice x Population interaction (F(1,651)=4.52, p=0.03,* $\eta_{p}$*²=0.01). However, Bayesian analysis indicates a* ${BF}_{incl}$*=0.4 in favor of H0). Post-hoc analyses indicated that all comparisons were significant excepted the obedience to send a shock between the two populations (t=-0.62, df=768, p=0.54). No other interaction was significant nor supporting H1 (all ps>0.50, all* ${BF}_{incl}$*<3)”.*

Note to the editor:

We realized that we made an error in the description of the MRI scanner used for this study. We also omitted to acknowledge an important contributor in the acknowledgments. We are therefore taking this second round of revision as an opportunity to correct both mistakes.

We corrected in the method section of the manuscript:

Lines 340 to 342: *“MRI images were recorded using a research-dedicated hybrid 3.0-Tesla SIGNA Positron Emission Tomography-Magnetic Resonance (PET-MR) scanner (GE Healthcare, Milwaukee, Wisconsin, USA) and a 24-channel head and neck coil, at Erasme Hospital in Brussels (Belgium)”.*

We added at the end of the manuscript an Acknowledgment section:

Lines 914 to 916: *“****Acknowledgements.*** *The project, as part of the PET-MR project at the Hôpital Universitaire de Bruxelles (Hôpital Erasme) is financially supported by the Association Vinçotte Nuclear (AVN, Brussels, Belgium)”.*
